# Electron-Induced Decomposition of Different Silver(I) Complexes: Implications for the Design of Precursors for Focused Electron Beam Induced Deposition

**DOI:** 10.3390/nano12101687

**Published:** 2022-05-15

**Authors:** Petra Martinović, Markus Rohdenburg, Aleksandra Butrymowicz, Selma Sarigül, Paula Huth, Reinhard Denecke, Iwona B. Szymańska, Petra Swiderek

**Affiliations:** 1 Institute for Applied and Physical Chemistry (IAPC), Fachbereich 2 (Chemie/Biologie), University of Bremen, Leobener Str. 5 (NW2), 28359 Bremen, Germany; petra@uni-bremen.de (P.M.); markus.rohdenburg@uni-leipzig.de (M.R.); selma.sariguel@studium.uni-hamburg.de (S.S.); 2 Wilhelm-Ostwald-Institute for Physical and Theoretical Chemistry (WOI), Leipzig University, Linnéstr. 2, 04103 Leipzig, Germany; che09dup@studserv.uni-leipzig.de (P.H.); denecke@uni-leipzig.de (R.D.); 3 Faculty of Chemistry, Nicolaus Copernicus University in Toruń, Gagarina 7, 87-100 Toruń, Poland; aleksandra.butrymowicz@doktorant.umk.pl (A.B.); pola@umk.pl (I.B.S.)

**Keywords:** focused electron beam induced deposition, silver precursors, precursor design, electron-induced chemistry, electron-stimulated desorption

## Abstract

Focused electron beam induced deposition (FEBID) is a versatile tool to produce nanostructures through electron-induced decomposition of metal-containing precursor molecules. However, the metal content of the resulting materials is often low. Using different Ag(I) complexes, this study shows that the precursor performance depends critically on the molecular structure. This includes Ag(I) 2,2-dimethylbutanoate, which yields high Ag contents in FEBID, as well as similar aliphatic Ag(I) carboxylates, aromatic Ag(I) benzoate, and the acetylide Ag(I) 3,3-dimethylbutynyl. The compounds were sublimated on inert surfaces and their electron-induced decomposition was monitored by electron-stimulated desorption (ESD) experiments in ultrahigh vacuum and by reflection−absorption infrared spectroscopy (RAIRS). The results reveal that Ag(I) carboxylates with aliphatic side chains are particularly favourable for FEBID. Following electron impact ionization, they fragment by loss of volatile CO_2_. The remaining alkyl radical converts to a stable and equally volatile alkene. The lower decomposition efficiency of Ag(I) benzoate and Ag(I) 3,3-dimethylbutynyl is explained by calculated average local ionization energies (ALIE) which reveal that ionization from the unsaturated carbon units competes with ionization from the coordinate bond to Ag. This can stabilise the ionized complex with respect to fragmentation. This insight provides guidance with respect to the design of novel FEBID precursors.

## 1. Introduction

Silver(I) carboxylates have lately received increasing attention regarding their application in focused electron beam induced deposition (FEBID) [1,2,3]. FEBID is a direct-write method, which is performed in electron microscopes and generates precisely defined metal nanostructures through electron-induced decomposition of suitable precursor molecules [4]. Gaseous precursors are injected into the vacuum chamber of the microscope, physisorb on a substrate surface, and are then dissociated under the focused electron beam. Ideally, this leads to pure metal deposits while volatile organic compounds are pumped away [4]. Unfortunately, the organic ligands often become incorporated in the deposits leading to unwanted contaminations. Without applying post-deposition purification methods, the metal content of typical FEBID deposits fabricated from metal-containing precursors typically lies in the range 5–40 atom% [3,5,6]. This impedes possible applications requiring, e.g., high electrical conductivity.

The first reported deposition of silver (Ag) via FEBID used the carboxylate complex Ag(I) 2,2-dimethylbutanoate ([Ag_2_(μ-O_2_CC(CH_3_)_2_C_2_H_5_)_2_]) as precursor [7]. Ag FEBID nanostructures are of interest because pure Ag is suitable for plasmonic applications due to small optical losses in the visible regime [7]. Carboxylate complexes have been successfully used in chemical vapour deposition (CVD) resulting in high purity thin metal films [8,9,10,11,12]. FEBID of Ag from Ag(I) 2,2-dimethylbutanoate was enabled by a fully integrated and heated gas injection system (GIS) that can handle Ag compounds despite their relatively low vapour pressures [7]. The results have revealed that Ag(I) 2,2-dimethylbutanoate is a promising FEBID precursor that produces high purity deposits with Ag contents of up to 73 atom% [7]. Recently, the fluorinated complex Ag(I) pentafluoropropionate ([Ag_2_(μ-O_2_CC_2_F_5_)_2_]) was also used for FEBID resulting in deposits with high silver contents of up to 76 atom% [3].

**Figure 1:**
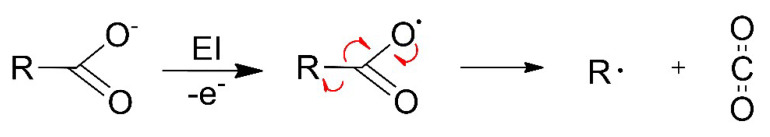
Fragmentation of carboxylate ligands in metal complexes initiated by ionization following electron impact, as proposed earlier [14,15,16,17]. Red arrows indicate the electron rearrangement that leads to release of CO_2_ and an organic radical R^•^.

The design of suitable FEBID precursors requires insight into the mechanisms that govern their electron-induced decomposition [13]. As demonstrated herein for the case of Ag(I) complexes, the efficient decomposition of the precursors depends decisively on the structure of the organic ligands that are coordinated to the metal. In the case of carboxylate ligands, the electron-induced fragmentation is thought to be driven by electron ionization (EI) leading to release of thermodynamically stable and volatile CO_2_ (Figure 1). This was deduced previously from studies on the electron-induced decomposition of thin surface layers of the coordination polymer Cu(II) oxalate and of the metal organic framework HKUST-1 [14,15], the latter being a three-dimensional coordination polymer consisting of Cu^2+^ ions that are linked by trivalent benzene-1,3,5-tricarboxylate anions. However, it was also observed that the removal of elements other than the metal during electron irradiation was much more efficient for Cu(II) oxalate than in the case of HKUST-1 [14], underlining that the loss of CO_2_ from the carboxylate is not the only factor that governs the efficiency of decomposition. In fact, it was proposed that the facile decomposition of Cu(II) oxalate is related to a reaction pathway that can convert the entire oxalate unit to the stable volatile product molecule CO_2_ [16].

Aiming at design strategies for precursors to be used in FEBID of Ag nanostructures, we report here surface science studies conducted in a clean ultrahigh vacuum (UHV) environment that investigate the electron-induced decomposition of Ag(I) complexes with different ligands. Volatile compounds released upon electron impact from a precursor layer were analysed by use of mass spectrometry (MS) in an electron-stimulated desorption (ESD) experiment. Complementarily, reflection–absorption infrared spectroscopy (RAIRS) was employed to monitor chemical changes in the precursor layer upon electron exposure. Although the studies were conducted with much lower primary electron energies (herein 50 eV, exceptionally 100 or 500 eV) than typically used in FEBID (>1 keV) [4], it is widely accepted that low-energy secondary electrons drive much of the precursor fragmentation that occurs under the electron beam [13,18,19,20]. Therefore, the present approach can reveal the typical decomposition reactions that are also relevant to the actual FEBID process.

**Figure 2:**
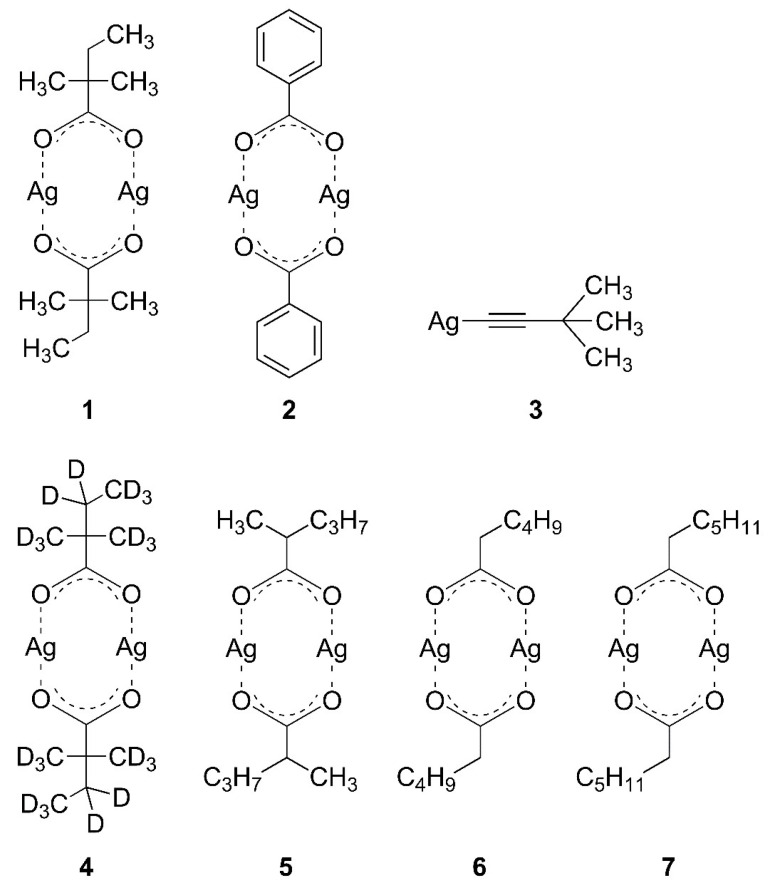
Silver(I) complexes used in the present study: Ag(I) 2,2-dimethylbutanoate (**1**); Ag(I) benzoate (**2**); Ag(I) 3,3-dimethyl-1-butynyl (**3**); Ag(I) 2,2-dimethylbutanoate-d_11_ (**4**); Ag(I) 2-methylpentanoate (**5**); Ag(I) hexanoate (**6**); Ag(I) heptanoate (**7**). The volatile Ag(I) carboxylate complexes (**1**,**2**,**4–7**) have a dimeric structure [21], while Ag(I) 3,3-dimethyl-1-butynyl (**3**) has been described as a polymeric material [22] but is shown here in its monomeric form for simplicity.

**Figure 3:**
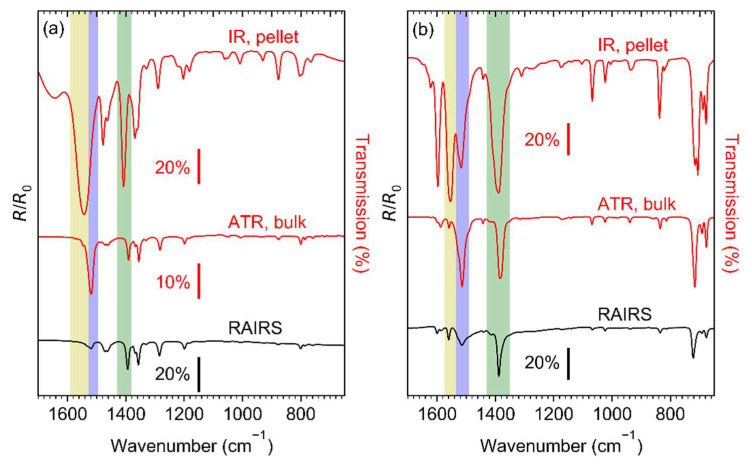

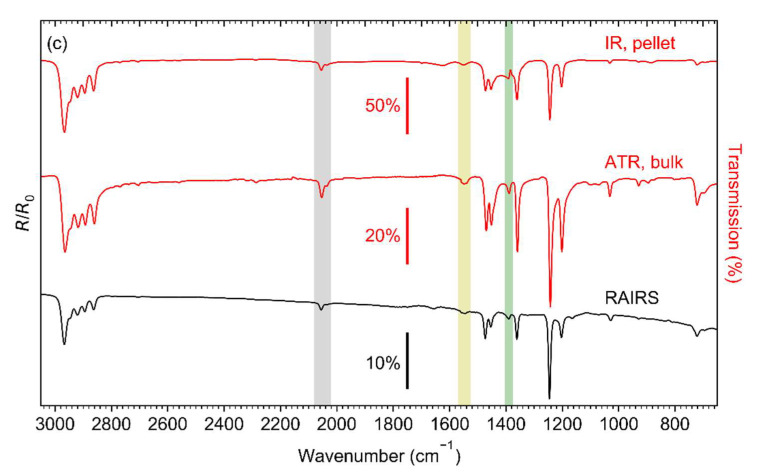
Infrared spectra of (**a**) Ag(I) 2,2-dimethylbutanoate, (**b**) Ag(I) benzoate, and (**c**) Ag(I) 3,3-dimethyl-1-butynyl. The two upper spectra in each frame were recorded in transmission from KBr pellet (IR) and by attenuated total reflectance (ATR) from the as-synthesised compound. The bottom spectra (RAIRS) were recorded in reflection from the compounds sublimated onto cl-BPT/Au substrates. The coloured bands highlight particular vibrational modes: *ν_as_*(COO^−^) in monodentate coordination (yellow), *ν_as_*(COO^−^) in bridging coordination (blue), *ν_s_*(COO^−^) (green), *ν*(≡CH) (grey).

We studied, in particular, the electron-induced decomposition of Ag(I) 2,2-dimethylbutanoate (Figure 2, (**1**)) as an example of a precursor that performs well in FEBID and compared its electron-induced decomposition to a carboxylate complex with aromatic side group, namely, Ag(I) benzoate (Figure 2, (**2**)), and an organometallic acetylide compound with a Ag-C bond, Ag(I) 3,3-dimethyl-1-butynyl (Figure 2, (**3**)). Furthermore, a fully deuterated Ag(I) 2,2-dimethylbutanoate and several Ag(I) carboxylates with different alkyl groups (Figure 2, (**4**–**7**)) were included to support the conclusions. Note that the volatile Ag(I) carboxylate complexes have a dimeric structure, as shown in Figure 2 [21], while Ag(I) 3,3-dimethyl-1-butynyl (3) has been described as a polymeric material [22] but is represented in Figure 2 by its monomeric form. Samples were prepared by sublimating these compounds onto self-assembled monolayers (SAMs) of 4-biphenylthiol (BPT) on Au surfaces that were fully crossed-linked by electron irradiation prior to the sublimation. The crossed-linked BPT (cl-BPT) SAM does not, by itself, give rise to ESD signals and suppresses unwanted adsorption of vapours on the Au support during handling of the samples under ambient conditions [23]. This approach thus ensures that ESD stems entirely from the sublimate layers. The results reveal that the ligands of Ag(I) 2,2-dimethylbutanoate and other compounds containing alkylated carboxylate ligands can be converted to volatile products by electron irradiation, while this is not the case for the aromatic carboxylate ligand and the alkynyl ligand. This work thus provides guidance for the choice of suitable Ag(I) carboxylate precursors for FEBID.

## 2. Materials and Methods

### 2.1. Precursor Synthesis

Ag(I) carboxylates (Ag(I) 2,2-dimethylbutanoate, Ag(I) benzoate, Ag(I) 2,2-dimethylbutanoate-d_11_, Ag(I) 2-methylpentanoate, Ag(I) hexanoate, and Ag(I) heptanoate) were synthesised from the corresponding carboxylic acids (2,2-dimethylbutanoic acid (Alfa Aesar, Haverhill, MA, USA, 97%)), benzoic acid (Merck, Darmstadt, Germany, 99%), 2,2-dimethylbutanoic acid d_11_ (CDN Isotopes, QC, Canada, 98.4% d_11_), 2-methylpentanoic acid (Sigma-Aldrich, St. Louis, MO, USA, 98%), hexanoic acid (Alfa Aesar, Haverhill, MA, USA, 98+%), heptanoic acid (SAFC, St. Louis, MO, USA, 97%)), and silver nitrate (Alfa Aesar, Haverhill, MA, USA, 99.9+%) using a simple precipitation reaction modified from a protocol reported previously [21]. A solution of the carboxylic acid (3.5 mmol) and potassium hydroxide (Riedel-de-Haën, Seelze, Germany, >85%, 3.5 mmol) in a water–ethanol mixture (1:1 *v*/*v*) was stirred and an equal volume of a solution of silver nitrate in water (3.5 mmol) was slowly added. A white precipitate formed, which was filtered, washed with water and ethanol, and dried in vacuo. Ag(I) 3,3-dimethyl-1-butynyl was obtained from Oliver Feddersen Clausen (www.modularflow.com, accessed on 9 November 2021) and used as received.

### 2.2. Sample Preparation

In line with experiments reported previously [23] and except for samples used in XPS experiments, all Ag(I) compounds were sublimated on a cl-BPT SAM, which was grown beforehand on an Au surface (200 nm Au on 5 nm Ti on boron-doped silicon, Georg Albert PVD). Prior to the preparation of the SAM, the Au surface was cleaned by immersion in peroxymonosulfuric acid (H_2_SO_4_/H_2_O_2_ 3:1 *v*/*v*), followed by rinsing with distilled water and ethanol. Each cleaning step was conducted for at least 15 min using ultrasonication. The cleaned Au surface was immersed in a 1 mM solution of BPT (Sigma-Aldrich, St. Louis, MO, USA, 97%) in ethanol for 72 h and then washed with ethanol and dried in a nitrogen stream. RAIRS confirmed the formation of the BPT SAM. For cross-linking, the BPT SAM was exposed to an electron dose of 40,000 µC/cm^2^ at E_0_ = 500 eV. This was sufficient to terminate desorption of H_2_ according to MS. Sublimation was conducted using a glassware sublimation apparatus. The cl-BPT-SAM was mounted on the coldfinger of the apparatus and the Ag(I) compound (amounts ranging from 1 to 15 mg depending on the compound) was filled into the flask. Sublimation was performed by evacuating the flask to 5 × 10^−2^ mbar and heating it to 200 °C (240 °C for Ag(I) benzoate) while the coldfinger was flushed with cooling water throughout the sublimation process. Samples inspected by optical microscopy and by XPS were prepared by sublimating the Ag(I) compounds onto cleaned Au substrates without SAM. All sublimated samples were characterised using RAIRS.

### 2.3. Electron-Stimulated Desorption (ESD) Experiments

ESD experiments were performed in a home-built UHV chamber described before [14,16]. The setup is equipped with a flood gun (SPECS FG15/40, SPECS Surface Nano Analysis GmbH, Berlin, Germany) for electron exposure and a quadrupole mass spectrometer (QMS) residual gas analyser (RGA) (Stanford Research Systems, Sunnyvale, CA, USA) with mass range *m*/*z* 1–300 for analysing the desorbing products. The electron gun delivers a divergent electron beam with a tunable energy in the range of 1–500 eV and with current densities measured on the sample between 10 and 30 µA/cm^2^. The setup consists of a main chamber and a small transfer chamber that can be evacuated and vented separately and is used to insert the sublimate samples. The samples were mounted on Cu sample holders, which were cleaned beforehand in the main chamber by electron irradiation at E_0_ = 500 eV. After inserting the samples, the transfer chamber was evacuated to a pressure of 1 × 10^−8^ mbar, allowing for sample transfer into the main chamber by use of a linear manipulator. The pressure in the main chamber was constantly kept below 5 × 10^−9^ mbar. To further reduce ESD from the Cu sample holder, the sample holders were covered by a Cu mask, which was kept in vacuum between experiments and exposes approximately 2 cm^2^ of the sample surface to the electron beam. The mask is electrically insulated from the sample and is held at chamber ground potential.

Most ESD experiments were carried out using an electron energy of E_0_ = 50 eV. Selected experiments were, in addition, performed at 100 or 500 eV. To accurately measure the sample current and therefore the electron exposure, a positive bias was applied to the sample. This prevents secondary electrons, which are produced upon irradiation, from escaping. For 50 eV (100 eV, 500 eV), the flood gun energy was set to 45 eV (90 eV, 480 eV) while a positive bias of +5 eV (+10 eV, +20 eV) was applied. The ESD experiments were performed at room temperature. The desorbing neutral products, which have desorption cross-sections that are typically orders of magnitude higher than those of ions [24], were analysed by MS in a selected *m*/*z* range after EI at 70 eV in the ion source of the QMS. The QMS was also used to measure mass spectra of 2-methyl-2-butene, CO_2_ and H_2_O, which were needed as reference to fit the ESD data of Ag(I) 2,2-dimethylbutanoate. Note that the RGA employed in this study discriminates against high *m*/*z* ratios, which impedes the usage of listed reference MS data [17].

### 2.4. Reflection–Absorption Infrared Spectroscopy (RAIRS)

RAIRS was used to confirm the successful sublimation of the Ag(I) compounds on cl-BPT SAMs and to monitor their decomposition in an ex situ experiment after electron exposure. RAIR spectra were measured as described previously [25] with an evacuated FTIR spectrometer (IFS 66v/S, Bruker Optics GmbH, Ettlingen, Germany) by accumulating 400 scans in the range between 4000 and 750 cm^−1^ with a resolution of 4 cm^-1^ using an aperture of 2.0 mm. The spectrometer is equipped with a grazing incidence reflection unit and a liquid nitrogen-cooled MCT detector with sufficient sensitivity down to 750 cm^−1^. During measurements, the sample chamber was evacuated to 5–8 mbar and the system was purged with N_2_ to eliminate residual vapours such as CO_2_ and H_2_O. A fully deuterated hexadecanthiol (HDT) SAM grown on a Au surface was used to measure background spectra.

### 2.5. Attenuated Total Reflection (ATR) and Transmission Infrared Spectroscopy

ATR infrared spectroscopy (ATR–IR) [26] was used to verify the integrity of the Ag(I) precursors prior to sublimation. The spectra were measured using a FTIR Spectrometer (Nicolet™ Summit, Thermo Scientific™, Waltham, MA, USA) with a monolithic germanium ATR crystal by accumulating 16 scans in the range between 4000 and 550 cm^−1^. In addition, transmission infrared spectra were measured on the evacuated FTIR spectrometer that was also used for RAIRS. For this, a mixture containing 0.1% of the Ag(I) compound and 99.9% KBr were pressed into a pellet. A pure KBr pellet was used as background.

### 2.6. Optical Microscopy

Optical microscopy (×40) was used to take photos of sublimated layers of Ag(I) 2,2-dimethylbutanoate, Ag(I) benzoate, and Ag(I) 3,3-dimethyl-1-butynyl. A Motic BA210 LED microscope (Motic, Barcelona, Spain) was used for this purpose.

### 2.7. X-Ray Photoelectron Spectroscopy

All XPS measurements were performed with a VG ESCALAB 220I XL spectrometer (Thermo Scientific™, Waltham, MA, USA) at room temperature and with a background pressure < 1 × 10^−8^ mbar. To excite the photoelectrons, non-monochromatised Al *K*_α1,2_ (1486.6 eV) radiation was used. The samples were attached to the sample holder by double-sided carbon tape, which also provides conducting contact.

All measurements were carried out in normal emission. To focus the emitted electrons, a lens mode characterised by a small angular acceptance (≈±4°) and a large detection area (Ø 5 mm) was used. The hemispherical electron energy analyser was operated in constant analyser energy mode. The detail spectra were recorded with pass energy of 50 eV. The photoelectron current was accumulated over 30 ms, parallel in six channeltrons, with energy steps of 0.1 eV. Each range was scanned four times, using an alternating recording mode.

All XPS data were processed using Unifit software (Version 2022, Unifit Scientific Software GmbH, Leipzig, Germany) [27]. For all detail spectra, the excitation satellites were subtracted. A charge correction was applied by setting the major C 1 s signal to a binding energy of 285 eV. As background function, the sum of a first order polynomial and Shirley function was used. The spectra were fitted using Voigt profiles. Within one spectrum, the Gaussian and Lorentzian widths were kept the same for all signals as well as doublet separations, if applicable. Peak height ratios within doublets were kept fixed, respecting the quantum mechanically derived intensity ratios.

### 2.8. Computational Methods

DFT-based geometry optimisation of Ag(I) compounds was carried out using the Gaussian16 software package (rev. C.01, Gaussian, Inc., Wallingford, CT, USA) [28]. The B3LYP/def2-TZVPP [29,30,31,32] model chemistry was employed including empirical dispersion corrections according to Grimme’s D3 method [33] involving Becke–Johnson damping (GD3BJ) [34]. The location of minimum structures on the potential energy surface was verified by the absence of imaginary frequencies in vibrational analyses. The average local ionization energy (ALIE) [35] was calculated in a three-dimensional grid out of the DFT wavefunction with the Multiwfn software (Version 3.8, Tian Lu, Beijing, China) [36].

## 3. Results

### 3.1. Characterisation of Sublimated Precursor Layers

Prior to electron irradiation, the integrity of the Ag(I) compounds after sublimation was verified and the structure and thickness of the sublimate layers were characterised. IR spectra of Ag(I) 2,2-dimethylbutanoate, Ag(I) benzoate, and Ag(I) 3,3-dimethyl-1-butynyl are shown in Figure 3. RAIRS data for the sublimate layers are compared to spectra of the as-prepared compounds recorded by transmission IR spectroscopy in KBr pellets and by ATR–IR. For each compound, all data were obtained from material resulting from the identical synthesis batch. Full range spectra of Ag(I) 2,2-dimethylbutanoate and Ag(I) benzoate are presented in Figure S1 (Supplemental Materials) and the detailed assignment of the bands is summarised in Tables S1–S3 (Supplemental Materials).

The transmission IR spectra of Ag(I) 2,2-dimethylbutanoate (Figure 3a) and Ag(I) benzoate (Figure 3b) obtained from the as-synthesised compounds agree well with previous results [21,37,38,39]. However, the positions of the asymmetric carboxylate stretching vibration *ν_as_*(COO^−^) at 1544 and 1553 cm^−1^ are indicative of a dominant contribution of ligands with a monodentate coordination [40,41,42,43]. The transmission IR spectrum of Ag(I) benzoate exhibits an additional band at 1517 cm^−1^, which is characteristic of a bridging coordination [40,41,42,43]. Only the bridging coordination is observed in the ATR–IR and RAIR spectra of both Ag(I) 2,2-dimethylbutanoate and Ag(I) benzoate. This is consistent with the presence of Ag(I) dimers bridged by two carboxylate ligands as also deduced earlier from mass spectra of gaseous Ag(I) 2,2-dimethylbutanoate [21]. As all spectra for a particular compound shown in Figure 3 have been obtained from the same synthesis batch, we conclude that the presence of unidentate species in the transmission spectra results from a phase transition induced by the high pressure applied when preparing the KBr pellets. In contrast, the RAIRS data support that sublimation preserves the dimer structure.

**Figure 4:**
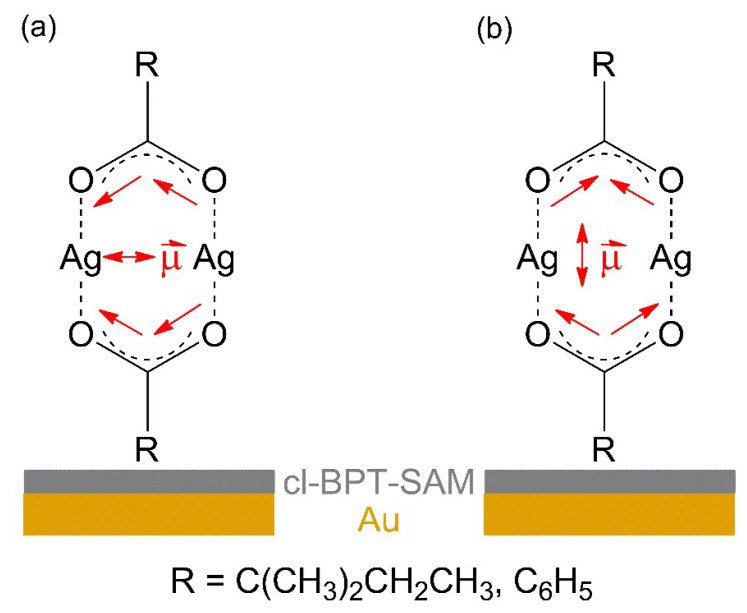
Qualitative model of the average arrangement of Ag(I) 2,2-dimethylbutanoate and Ag(I) benzoate when sublimated onto the cl-BPT/Au substrates as derived from RAIRS. For molecules standing near upright, the transition dipole moment (TDM) of is *ν_as_*(COO^−^) is close to parallel to the substrate leading to low intensity (**a**) and the TDM *ν_s_*(COO^−^) is near perpendicular to the substrate (**b**).

The intensity of *ν_as_*(COO^−^) is low as compared to *ν_s_*(COO^−^) in the RAIR spectra obtained from both carboxylate complexes (Figure 3a,b). In contrast, *ν_as_*(COO^−^) has a high intensity in ATR–IR. This indicates that despite the lack of specific binding sites on the cl-BPT SAM, the molecules assume on average a near-upright orientation (Figure 4). In this case, the transition dipole moment (TDM) of *ν_as_*(COO^−^) is close to parallel to the underlying Au surface and therefore the band has a low intensity according to the surface selection rule [44]. In contrast, the TDM of *ν_s_*(COO^−^) is near perpendicular to the surface so that this vibration is well visible.

All of the IR spectra obtained from Ag(I) 3,3-dimethyl-1-butynyl (Figure 3c) are in excellent agreement with those of free 3,3-dimethyl-1-butyne [45,46] except that coordination to Ag(I) ions is obvious from the missing CH stretching vibration of the acetylene unit *ν*(≡CH), which would be located at 3309 cm^−1^ (see Figure S1 and Table S3, Supplemental Materials). Note that the spectra show additional small and broader bands around 1550 and 1390 cm^−1^. These are the characteristic *ν_as_*(COO^−^) and *ν_s_*(COO^−^) bands of carboxylate ligands (compare Tables S1 and S2, Supplemental Materials) indicative of an impurity resulting from CO_2_ uptake. This is in line with previous reports that Ag(I) alkynes tend to incorporate CO_2_ into their Ag-C bond leading to formation of Ag(I) carboxylates [47].

At an electron energy of 50 eV, applied in most of the irradiation experiments shown in Section 3.2 and Section 3.3, the effective attenuation length of electrons in a material is typically of the order of 1 nm [48]. Sufficiently thin sublimate layers are thus needed to be able to fully decompose the sample by electron irradiation. As shown in Section 3.2, complete decomposition was achieved for sublimate layers of Ag(I) 2,2-dimethylbutanoate but not in the cases of Ag(I) benzoate and Ag(I) 3,3-dimethyl-1-butynyl. As the aim of this study is to relate the decomposition efficiency to the molecular structure of the Ag(I) complexes, we must rule out that incomplete decomposition results from a sublimate thickness that is beyond the penetration depth of the electron beam. Therefore, we used infrared spectroscopy (see Figure S2 and Table S4, Supplemental Materials) to establish that the amount of sublimate material in the samples of Ag(I) benzoate and Ag(I) 3,3-dimethyl-1-butynyl does not exceed that present in Ag(I) 2,2-dimethylbutanoate samples. Furthermore, we examined the homogeneity of the samples by visual inspection using optical microscopy (Figure S3, Supplemental Materials) and XPS (Table S5 and Figure S4, Supplemental Materials). It is not trivial to relate this information to a sublimate thickness because, as obvious from the microscopic images, the material is not homogeneously distributed on the supporting surface. In fact, formation of islands is often encountered when layers are grown from the gas phase [23]. Additionally, according to the RAIRS data (Figure S2), the actual amount of sublimate varies somewhat between individual samples. This most likely relates to slight variations in the sublimation conditions (temperature, pressure) from one experiment to the next. In particular, the flux and temperature of the cooling water could not be quantitatively controlled. However, as outlined in part 2 of the Supplemental Materials, the intensity of RAIRS spectra depends linearly on the amount of material that is probed within the thickness regime considered herein [25,49]. Based on this and on a quantitative analysis of the XPS intensities derived from reported procedures [50,51], we can safely conclude that the amount of material present in the sublimates of Ag(I) benzoate and Ag(I) 3,3-dimethyl-1-butynyl generally did not exceed the amount of material present in sublimates of Ag(I) dimethylbutanoate. This conclusion is important for the evaluation of the results presented in Section 3.2.

### 3.2. Electron-Induced Decomposition of Ag(I) 2,2-dimethylbutanoate, Ag(I) Benzoate, and Ag(I) 3,3-dimethyl-1-butynyl

**Figure 5:**
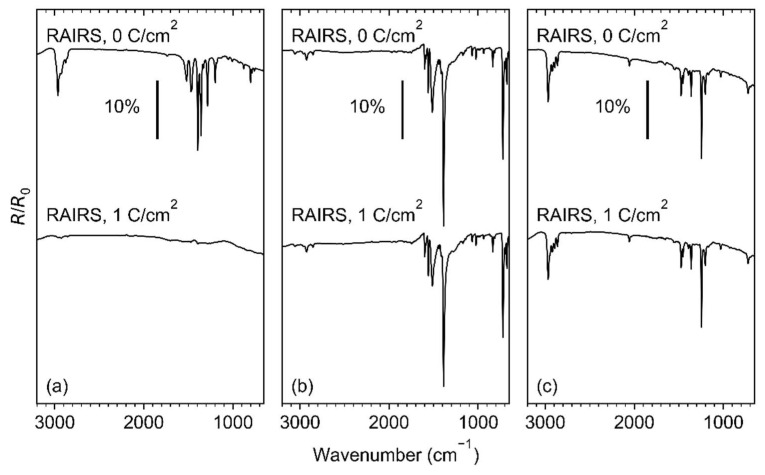
RAIR spectra of (**a**) Ag(I) 2,2-dimethylbutanoate and (**b**) Ag(I) benzoate, and (**c**) Ag(I) 3,3-dimethyl-1-butynyl before (0 C/cm^2^) and after irradiation (1 C/cm^2^) at E_0_ = 50 eV.

The effect of electron irradiation on the sublimated layers of Ag(I) 2,2-dimethylbutanoate, Ag(I) benzoate, and Ag(I) 3,3-dimethyl-1-butynyl was compared using RAIRS, as shown in Figure 5. A complete loss of the vibrational bands is observed in the case of Ag(I) 2,2-dimethylbutanoate (Figure 5a) after an electron exposure of 1 C/cm^2^ at 50 eV. RAIRS data recorded after increasing exposures and presented in Figure S5 (Supplemental Materials) reveal that an exposure of 1 C/cm^2^ is, in fact, needed for a complete conversion of the sublimate. In contrast, only a minor loss of intensity is observed for Ag(I) benzoate (Figure 5b) and Ag(I) 3,3-dimethyl-1-butynyl (Figure 5c), despite the somewhat lower amount of material in the sublimate (see Table S4, Supplemental Materials). This reveals a significantly lower sensitivity towards electron irradiation for the latter two compounds as compared to Ag(I) 2,2-dimethylbutanoate, despite a comparable or even lower average sublimate thickness.

**Figure 6:**
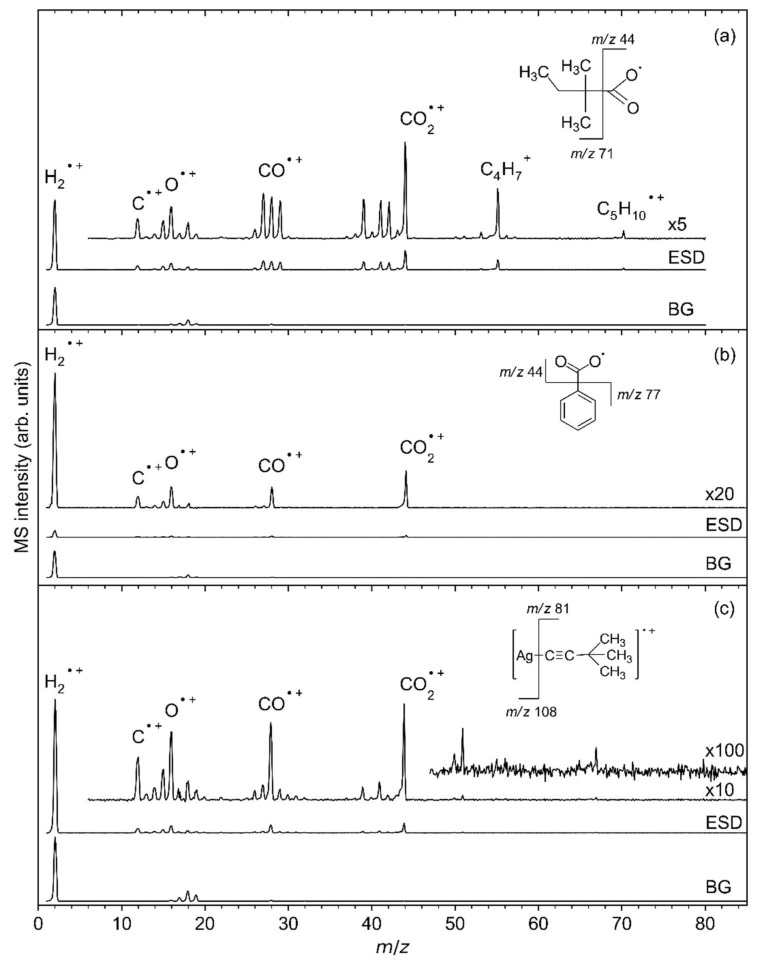
Mass spectra of the volatile species produced upon ESD (E_0_ = 50 eV) from (**a**) Ag(I) 2,2-dimethylbutanoate and (**b**) Ag(I) benzoate, and (**c**) Ag(I) 3,3-dimethyl-1-butynyl as well as background MS measured directly before each irradiation. All ESD MS were corrected by subtracting the respective background mass spectrum (BG). Note that the dominant fragments in (**c**) give evidence that a carboxylate impurity has formed in the Ag(I) 3,3-dimethyl-1-butynyl sample.

During irradiation, electron-stimulated desorption (ESD) of neutral species from the sublimate layers was monitored by EI–MS, which produces, in the QMS ion source, the cations and fragments thereof discussed below. Figure 6 shows mass spectra acquired during the initial stages of irradiation from the same samples of the three Ag(I) complexes, as presented in Figure 5. The ESD mass spectrum of Ag(I) 2,2-dimethylbutanoate (Figure 6a) reveals desorption of H_2_ and CO_2_ as indicated by the most intense signals at *m*/*z* 2 (H_2_^•^^+^) and *m*/*z* 44 (CO_2_^•^^+^). While some H_2_ is generally present as dominant background gas (see bottom curves in each frame of Figure 6), CO_2_ stems exclusively from the decomposition of the sublimate layer. Additional signals at *m*/*z* 70 (C_5_H_10_^•^^+^) and *m*/*z* 55 (C_5_H_10_^•^^+^-CH_3_^•^) that are also absent from the background gas are characteristic of desorption of a hydrocarbon compound and are thus accompanied by signals in the ranges of C_3_ fragments (*m*/*z* 39 (C_3_H_3_^+^) and *m*/*z* 41–43), C_2_ fragments (*m*/*z* 26–29), and C fragments (*m*/*z* 13–15 with *m*/*z* 15 assigned to CH_3_^+^). Further signals at *m*/*z* 16 (O^•^^+^) and *m*/*z* 12 (C^•^^+^) relate to the fragmentation pattern of CO_2_ (see below). The signal *m*/*z* 18 (H_2_O^•^^+^) gives evidence of a certain amount of humidity that is present, in particular, in the background gas of the vacuum chamber.

It is important to note that ESD was only observed for sufficiently thin sublimate layers. At larger sublimate thickness, the MS intensities decreased strongly while the RAIR spectrum changed to that also obtained by ATR–IR from the bulk material (Figure S6, Supplemental Materials). We take this as indication that sublimation has produced larger crystallites with size beyond the penetration depth of the electron beam. This most likely leads to charge accumulation which repels the impinging electrons leading to loss of ESD intensity.

**Figure 7:**
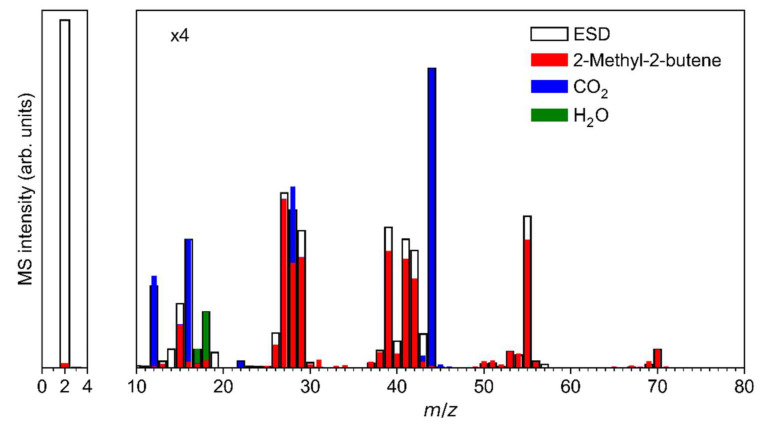
Reproduction of the mass spectrum (*m*/*z* 0–80) of the volatile species produced upon ESD (E_0_ = 50 eV) from Ag(I) 2,2-dimethylbutanoate from Figure 6a as a superposition of contributions from CO_2_, H_2_O, and 2-methyl-2-butene. The experimental ESD data were fitted by mass spectra obtained from the pure compounds: first, the spectrum of 2-methyl-2-butene was scaled to the ESD intensity at *m*/*z* 70; next, the spectrum of CO_2_ was added with scaling factor so that the ESD intensity at *m*/*z* 44 was reproduced; and last, the spectrum of H_2_O was added with scaling factor set to reproduce the ESD intensity of *m*/*z* 18.

**Figure 8:**
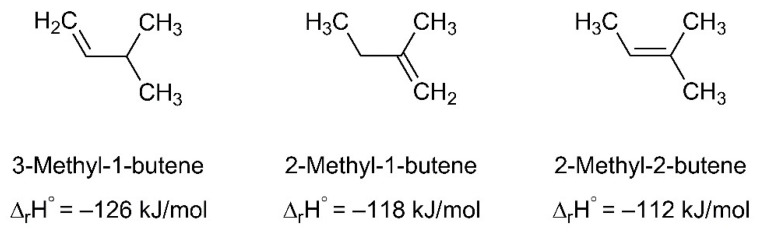
Different alkenes that can be formed after hydrogen radical loss from the C_5_H_11_^•^ radical produced upon electron irradiation of Ag(I) 2,2-dimethylbutanoate. The enthalpy of reaction at standard conditions (∆_r_H^°^) for hydrogenation of the double bond that yields the same final product C_5_H_12_ in each case indicates that 2-methyl-2-butene is the most stable among these isomers [53,54].

The ESD mass spectrum of Ag(I) 2,2-dimethylbutanoate (Figure 6a) was modelled by overlapping reference mass spectra of 2-methyl-2-butene, CO_2_ and H_2_O (Figure 7, see also Figure S7 in Supplemental Materials for individual reference MS data). The same QMS as for the ESD experiments was used to record reference mass spectra during leaking of the individual gases into the vacuum chamber. This provides for a more reliable analysis than the use of literature data because the sensitivity of the instrument decreases towards higher *m*/*z* ratios [17]. We note that three different alkenes can be formed by cleaving a hydrogen radical from the alkyl radical C_5_H_11_^•^ that is released upon loss of CO_2_ from Ag(I) 2,2-dimethylbutanoate (Figure 8). The mass spectra of these three C_5_H_10_ isomers are very similar [52] so that a unique assignment is not strictly possible. However, 2-methyl-2-butene was selected for the analysis of the ESD mass spectrum and we also refer to the desorbing product as such in the following discussion because it is the most stable of these isomers (see Figure 8). In this analysis, the intensity of the mass spectrum of 2-methyl-2-butene was first scaled so that the height of the *m*/*z* 70 signal matches the ESD mass spectrum. Next, the spectrum of CO_2_ was scaled and added so that the overall intensity of the *m*/*z* 44 signal in the ESD data was well reproduced. Finally, the same procedure was applied to H_2_O based on the *m*/*z* 18 signal. The resulting modelled mass spectrum provides a reasonable reproduction of the ESD mass spectrum (Figure 7) and thus supports that Ag(I) 2,2-dimethylbutanoate yields CO_2_ and 2-methyl-2-butene as most likely isomer of C_5_H_10_ under electron irradiation. Overall, this indicates that electron-induced fragmentation leads to loss of the entire alkyl side group, which would yield a C_5_H_11_^•^ radical. As obvious from our analysis, the radical converts to the stable olefin prior to desorption (see also Section 4). This latter reaction must proceed by loss of atomic hydrogen that can recombine to H_2_, thus explaining the increase in the *m*/*z* 2 signal under electron irradiation as compared to the residual gas background spectrum (Figure 6a).

We note that significant H_2_ desorption from the cl-BPT SAM can be ruled out because cross-linking was always performed until ESD of H_2_ had ceased (see Section 2.2). This was also verified by exposing a cl-BPT SAM to ambient conditions before it was reintroduced into UHV and further irradiated, which then resulted in negligible ESD of H_2_ (see Figure S8, Supplemental Materials). As a further test to confirm the origin of the H_2_ ESD signal, perdeuterated Ag(I) 2,2-dimethylbutanoate was also investigated. In this case, ESD recorded upon electron irradiation at 100 eV shows patterns that correspond closely to the nondeuterated analogue (Figure S9 and Table S6, Supplemental Materials). Note that the ESD pattern of the nondeuterated Ag(I) 2,2-dimethylbutanoate at 100 eV (Figure S9) agrees closely with the result for 50 eV. The deuterated compound in fact shows desorption of D_2_ (*m*/*z* 4) upon electron irradiation, which supports the hypothesis that atomic hydrogen (H or D) released from the C_5_H_11_^•^ (C_5_D_11_^•^) radical is the origin of 2-methyl-2-butene. However, D_2_ is accompanied by even stronger signals of HD (*m*/*z* 3) and H_2_ (*m*/*z* 2). Considering the high isotope purity (98.4%) of the acid used in the synthesis of perdeuterated Ag(I) 2,2-dimethylbutanoate and the lack of ESD from the supporting cl-BPT SAM, we relate the formation of HD and H_2_ to the electron-induced fragmentation of small quantities of H_2_O present as residual humidity (see above). This also indicates that H_2_O must contribute to ESD of H_2_ from nondeuterated Ag(I) 2,2-dimethylbutanoate.

Note that differently branched alkyl chains on the carboxylate ligands show analogous fragmentation behaviour. In all isomers of Ag(I) 2,2-dimethylbutanoate but also in the case of Ag(I) heptanoate, desorption of an olefin resulting from loss of CO_2_ and an additional H atom is observed in ESD performed at 100 eV (Figure S10, Supplemental Materials). Additionally, the ligands are again completely lost within an exposure of 1 C/cm^2^ according to RAIRS (Figure S11, Supplemental Materials).

In the case of Ag(I) benzoate (Figure 6b), the ESD intensity is considerably smaller than for Ag(I) 2,2-dimethylbutanoate. While the lower intensity as such may relate to the somewhat lower amount of sublimate, it is remarkable that only H_2_, CO_2_, traces of H_2_O, and possibly some CO desorb under electron irradiation. In contrast, desorption of products that relate to the phenyl (C_6_H_5_) group of the benzoate ligand is absent. This indicates that, in contrast to carboxylate ligands with alkyl groups, the aromatic ring is not converted to a volatile product after electron-induced loss of CO_2_. The origin of this different behaviour is discussed in Section 4.

Finally, ESD of Ag(I) 3,3-dimethyl-1-butynyl (Figure 6c) is dominated again by desorption of CO_2_ showing the same characteristic signals at *m*/*z* 44, 28, 16, and 12. This is unexpected from the molecular structure of this compound but in line with the carboxylate infrared bands seen in RAIRS of thin sublimate layers (Figure 3c). ESD also shows small hydrocarbon signals. A fragment with *m*/*z* 81 that represents the mass of the entire ligand is, in addition, visible in the case of a thicker sublimate layer irradiated at higher electron energy (Figures S12 and S13, Supplemental Materials). However, based on the particularly small loss of intensity in RAIRS of the thinner sublimate under electron exposure (Figure 5c) and considering previous reports that the elemental composition of Ag(I) 3,3-dimethyl-1-butynyl powder did not change under electron irradiation in an electron microscope [7], we propose that ESD of the 3,3-dimethyl-1-butynyl ligand rather stems from molecules that have reacted with CO_2_ from the ambient during sample handling than from pristine Ag(I) 3,3-dimethyl-1-butynyl. The obvious lack of sensitivity of Ag(I) 3,3-dimethyl-1-butynyl towards electron irradiation is also discussed further in Section 4.

### 3.3. Kinetics of ESD from Ag(I) 2,2-dimethylbutanoate

**Figure 9:**
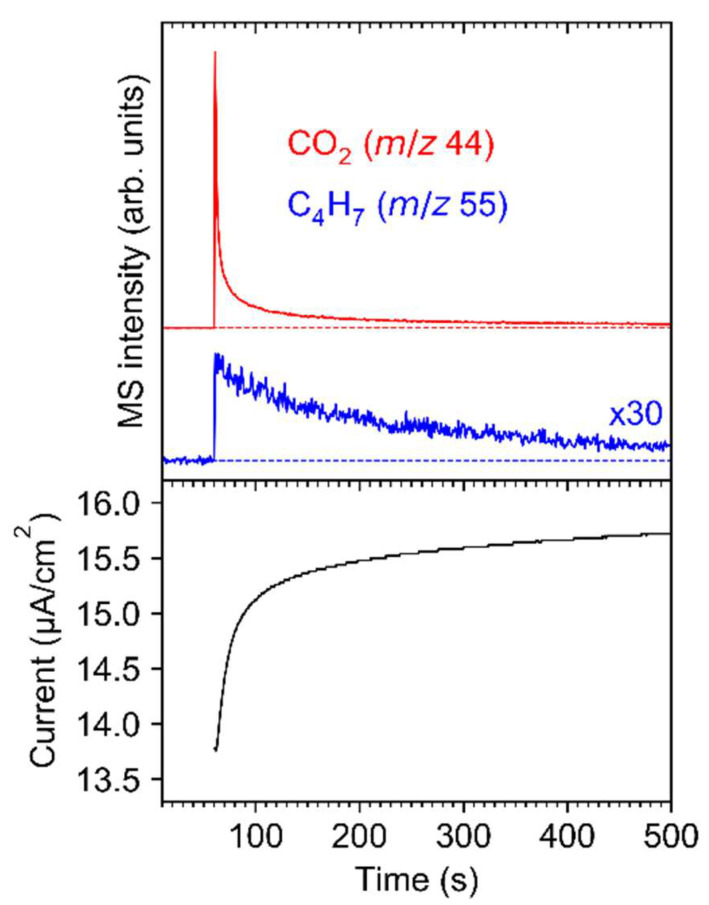
ESD as function of time during electron irradiation of Ag(I) 2,2-dimethylbutanoate at E_0_ = 50 eV monitored for *m*/*z* 44 (top panel, red, representative of CO_2_) and *m*/*z* 55 (top panel, blue, representative of 2-methyl-2-butene). The sudden steep increase in the ESD signals marks the start of irradiation. During irradiation, the current was measured on the sample (bottom panel), reaching a value of 16.46 µA/cm^2^ after a total exposure of 0.4 C/cm^2^.

Further insight into the electron-induced decomposition of Ag(I) 2,2-dimethylbutanoate upon irradiation with an energy of 50 eV was obtained by recording the evolution of ESD signals with characteristic *m*/*z* ratios as function of time. Note that RAIRS data for the samples described in this section are included in Figure S2 and Table S4 (Supplemental Materials). The experiments monitored *m*/*z* 44 that relates to desorption of CO_2_ and *m*/*z* 55, representative of 2-methyl-2-butene or its isomers. Both signals increase abruptly at the start of irradiation followed by a decay, which is particularly steep in the case of CO_2_ (Figure 9, top). During this decay, the current that is transmitted through the sample (Figure 9, bottom) increases slowly but steadily. This effect can result from depletion of the sublimate layer and thus increased transmission of electrons to the substrate or from trapping of some positive charge in the layer that accelerates the electrons towards the sample. However, the increasing current indicates that the simultaneous decrease in the ESD intensity does not result from deflection of the electron beam due to negative charging of the sample but must relate to the decomposition kinetics of the sample. Notably, the desorption rate of 2-methyl-2-butene decreases more slowly than that of CO_2_. This supports that CO_2_ is released via the initial electron-induced fragmentation while 2-methyl-2-butene is formed in a subsequent and somewhat slower reaction step that involves loss of atomic hydrogen.

**Figure 10:**
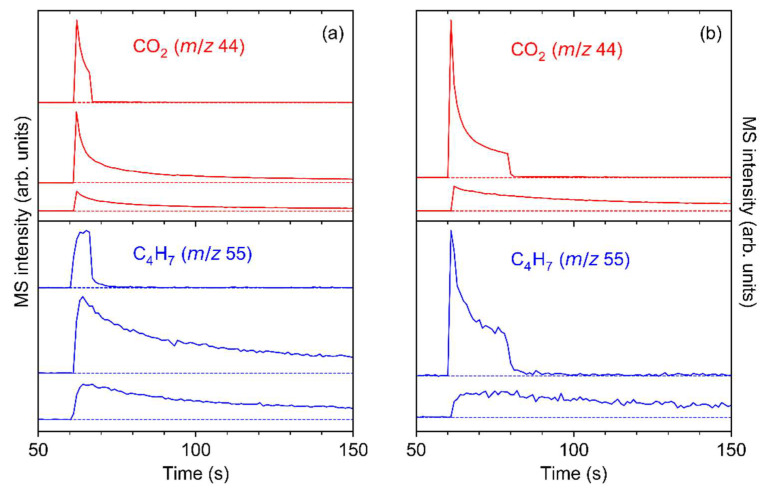
ESD as function of time during electron irradiation of Ag(I) 2,2-dimethylbutanoate at E_0_ = 50 eV for *m*/*z* 44 (top of panel, red, representative of CO_2_) and *m*/*z* 55 (bottom of panel, blue, representative of 2-methyl-2-butene). (**a**) The irradiation was interrupted after an exposure of 60 μC/cm^2^ (first row, average current 14.0 μA/cm^2^) and 1400 μC/cm^2^ (second row, average current 17.0 μA/cm^2^) before the last exposure of 100,000 µC/cm^2^ (third row, average current 16.9 µA/cm^2^). In between irradiations, the sample was retrieved from UHV for RAIRS. (**b**) The irradiation was interrupted for 90 min after 180 μC/cm^2^ (first row, average current 13.0 μA/cm^2^) and the sample left in UHV until irradiation was resumed with an exposure of 400,000 µC/cm^2^ (second row, average current 17.1 µA/cm^2^). Except for the first row, the graphs are clipped to the initial period of irradiation for better visualisation.

The experiment was repeated with several individual samples to confirm the different ESD kinetics for CO_2_ and 2-methyl-2-butene. Figure 10 shows two experiments where the irradiation was interrupted to reveal effects of possible aging of the sample upon contact to ambient conditions during transfer from UHV to RAIRS (Figure 10a) or in UHV (Figure 10b). In both cases, a slower decay or even an initial slight increase in the ESD signal of 2-methyl-2-butene was observed after the start of irradiation, in line with the result of the experiment shown in Figure 9. This effect was also observed in the case of an additional experiment performed at electron energy of 500 eV (Figure S14, Supplemental Materials). Note that variations in the time-dependent ESD curves between samples possibly relate to the morphology of the individual samples that may exhibit different crystallite sizes due to fluctuations of the temperature during sublimation. A more detailed investigation of this effect was, however, beyond the scope of the present work.

Figure 10 also reveals that irradiated sublimate layers of Ag(I) 2,2-dimethylbutanoate are subject to further chemical modification when handled in ambient conditions. For the sample that remained in UHV when electron exposure was interrupted, ESD resumed with similar intensity as seen at the end of the first electron exposure when the irradiation was switched on again (Figure 10b). In contrast, when the sample was exposed to ambient conditions prior to the next irradiation, the ESD intensity for both *m*/*z* 44 and *m*/*z* 55 was significantly higher than at the end of the previous irradiation (Figure 10a). This indicates that electron irradiation activates the sublimate towards reaction with constituents of air. This effect may be akin to the uptake of CO_2_ by Ag(I) 3,3-dimethyl-1-butynyl, as discussed in Section 3.1. In line with a previous theoretical study [55], we tentatively propose that Ag^+^ ions remaining in the sublimate layer after electron-induced fragmentation of Ag(I) 2,2-dimethylbutanoate react with CO_2_ from the ambient atmosphere upon handling in air. When resuming electron irradiation, this additional CO_2_ is released in addition to that produced from the remaining intact Ag(I) compound.

Finally, we compare the time scales for decomposition of Ag(I) 2,2-dimethylbutanoate as observed in ESD and RAIRS. As can be estimated from Figure 9 and Figure 10, the CO_2_ ESD signal decays to a level near the baseline within an electron exposure of the order of a few 10 mC/cm^2^ at an electron energy of 50 eV. In contrast, an electron exposure of the order of 0.1 C/cm^2^ is required for the same sample to achieve a visible reduction in the RAIRS intensities (Figure S15, Supplemental Materials). The rapid decay of the CO_2_ ESD signal is similar to earlier results for Cu(II) oxalate and related coordination polymers grown on a surface in a layer-by-layer process [16]. On the other hand, exposures of an order of only 10 mC/cm^2^ were necessary for the RAIRS signals to disappear for the thinnest investigated layers of Cu(II) oxalate while the required exposures clearly increased with the thickness of the layers [16]. This general behaviour indicates that only the fragments produced in the uppermost layers of the materials desorb rapidly while diffusion from layers further from the vacuum interface and possibly also inelastic scattering of the electron beam within the layer limits the ESD process at later stages of the irradiation. However, as seen in Figure 5, sublimates of Ag(I) 2,2-dimethylbutanoate can in fact be completely decomposed when a sufficiently long electron exposure is applied.

## 4. Discussion

The ESD and RAIRS data presented above reveal that Ag(I) 2,2-dimethylbutanoate and similar aliphatic Ag(I) carboxylates are decomposed more efficiently under electron irradiation than Ag(I) benzoate and Ag(I) 3,3-dimethyl-1-butynyl. The different decomposition behaviour of the three types of compounds can result from (i) different fragmentation efficiencies upon electron impact and (ii) reactions that are specific to particular fragments released by the initial electron–molecule interaction. These two effects are discussed herein. Furthermore, reactions with ambient vapours prior to electron irradiation play a role in the decomposition Ag(I) 3,3-dimethyl-1-butynyl.

**Figure 11:**
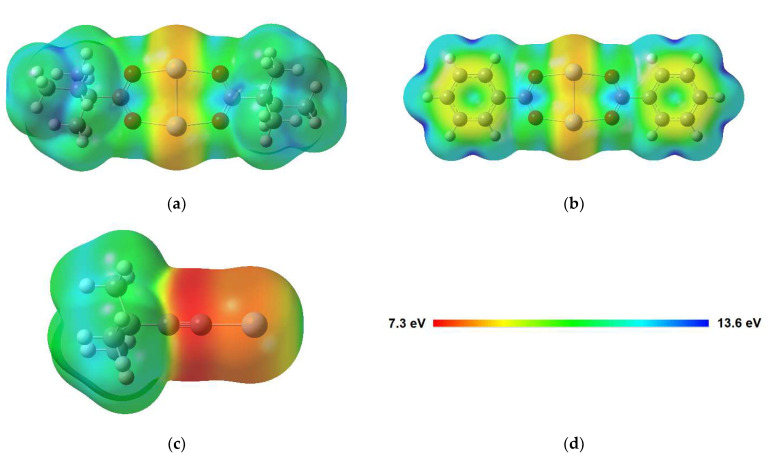
Average local ionization energy (ALIE) mapped onto the molecular surface (0.001 a.u. electron density isosurface) for (**a**) Ag(I) 2,2-dimethylbutanoate and (**b**) Ag(I) benzoate dimers, as well as (**c**) the monomer of Ag(I) 3,3-dimethyl-1-butynyl calculated at the B3LYP-GD3BJ/def2TZVPP level. (**d**) The colour code represents the distribution of ALIE in each molecule.

As proposed before [14,16,17], the electron-induced fragmentation of precursors with carboxylate ligands is most likely triggered by ionization (see Figure 1). The present results indicate that carboxylate ligands with saturated side chains are more efficiently removed from the sublimate layer than the aromatic benzoate ligand. This is reminiscent of earlier results for surface grown coordination polymers showing that CO_2_ is more efficiently released from Cu(II) oxalate than from HKUST-1 [14]. While the oxalate dianion consists of two carboxylate groups that are directly bound to each other, the trivalent linker of HKUST-1 contains an aromatic ring as also present in Ag(I) benzoate. The comparably low efficiency of CO_2_ loss from HKUST-1 and Ag(I) benzoate thus suggests that the aromatic ring stabilises the material with respect to fragmentation under electron irradiation. This effect is not included in the fragmentation mechanism represented in Figure 1, which assumes that ionization removes an electron from the negatively charged carboxylate group. To substantiate the hypothesis that the aromatic ring counteracts the depicted fragmentation channel, we investigated the average local ionization energy (ALIE) [56] of Ag(I) 2,2-dimethylbutanoate and Ag(I) benzoate, also including Ag(I) 3,3-dimethyl-1-butynyl as another example with unsaturated structural unit (Figure 11). For both carboxylate compounds, the ALIE reveals particularly low values at the Ag atoms in line with the coordinate bond donation that shifts electron density from the negatively charged carboxylate group to the positively charged Ag. Ionization thus leads preferably to removal of electron density from these coordinate bonds similar to the simplified model depicted in Figure 1. However, in the case of Ag(I) benzoate (Figure 11b), additional sites with relatively low values of the ALIE are localised on the aromatic ring. This points to an increased probability that ionization occurs from the hydrocarbon side group in Ag(I) benzoate. The possibility to delocalise the resulting charge over the ring is expected to counteract dissociation following ionization. This provides an explanation for the lower CO_2_ yield upon electron irradiation of Ag(I) benzoate as compared to Ag(I) 2,2-dimethylbutanoate where the ALIE is high on the alkyl side group. The calculation thus reveals that the side group of the carboxylate ligands can have an effect on the efficiency of the electron-induced fragmentation of a metal carboxylate complex.

Note that a similar fragmentation becomes possible when Ag(I) 3,3-dimethyl-1-butynyl reacts with CO_2_ under ambient conditions to form again a carboxylate complex (see Section 3.1 and Section 3.2). However, here again, concurrent ionization from the triple bond most likely lowers the fragmentation efficiency upon ionization. This also rationalises the low sensitivity of Ag(I) 3,3-dimethyl-1-butynyl itself towards electron irradiation [7]. In analogy to the situation in Ag(I) benzoate, ionization from the unsaturated CC triple bond of the ligand is most favourable. This is again visualised by the ALIE calculated for the monomeric structure of Ag(I) 3,3-dimethyl-1-butynyl (Figure 11c). The ALIE is particularly low, also compared to Ag(I) benzoate, on the triple bond. We note that the stability of Ag alkynyls is also supported by reports that such compounds can form under mild conditions when an alkynyl hydrocarbon is adsorbed onto a Ag(111) surface in the presence of O_2_ [57]. The persistence of the vibration *ν*(C≡C) under electron irradiation (Figure 5 and Table S3, Supplemental Materials) further indicates that ionization of Ag(I) 3,3-dimethyl-1-butynyl does not lead to polymerization as known in the case of olefins [58], which we attribute to steric hindrance by the bulky tertiary butyl group.

**Figure 12:**
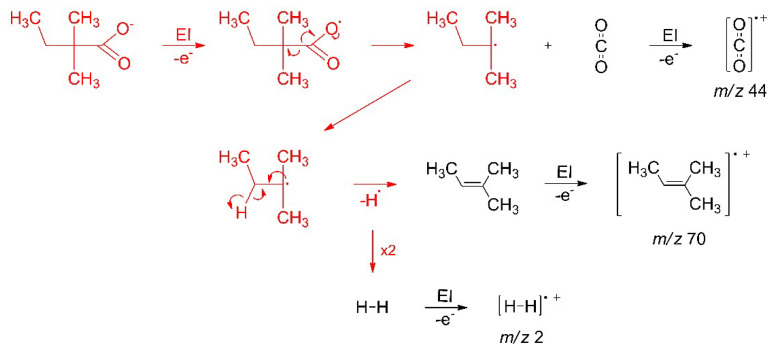
Mechanism of the electron-induced decomposition of Ag(I) 2,2-dimethylbutanoate to stable volatile reaction products. The electron-induced fragmentation and subsequent loss of atomic hydrogen from the radical fragment proceed on the surface (red) while stable volatile products desorb to be identified by their characteristic fragments formed after ionization in the ion source of the MS (black).

Molecular radical fragments that are released by the initial electron–molecule interaction can undergo different reactions depending on their structure and on reaction partners that may be available in their vicinity. The ESD results (Section 3.2) show that the radical ^∙^C_5_H_11_ released upon electron-induced loss of CO_2_ from Ag(I) 2,2-dimethylbutanoate converts to a stable and volatile olefin that can desorb and thereby remove carbon from a deposit during the FEBID process. Figure 12 summarises this sequence of reactions. In principle, radical species can also add to double bonds [17], but these are absent from the intact adjacent molecules. Additionally, the radical site carries bulky substituents that hinder the approach towards a reaction partner. Stabilisation by transfer or loss of hydrogen is thus apparently the most rapid reaction. We note that a saturated product resulting from recombination of the hydrogen radical with a second ^∙^C_5_H_11_ radical is not visible in the ESD data (Figure 6). This can be deduced from the lack of MS signals at *m*/*z* 57 and *m*/*z* 72 that are characteristic of a hydrocarbon C_5_H_12_ [52].

In contrast to the case of the saturated alkyl radical ^∙^C_5_H_11_ released from Ag(I) 2,2-dimethylbutanoate, stabilisation of the phenyl radical ^∙^C_6_H_5_ that results from expulsion of CO_2_ in the case of Ag(I) benzoate by loss of atomic hydrogen is not favourable. Such a reaction would lead to a highly strained ring structure with a triple bond. Recombination reactions between phenyl radicals are thus more likely to occur. This has been extensively reported in the case of BPT and related SAMs [59], and has also been applied here in the preparation of the cl-BPT SAMs used as support for the sublimate layers. The lack of signals relating to ESD of the phenyl ring or of a benzene molecule (C_6_H_6_) that would result from recombination of the phenyl radical ^∙^C_6_H_5_ with a hydrogen radical released from another molecule under electron irradiation strongly supports that cross-linking is also the most likely reaction of ^∙^C_6_H_5_ in the sublimate layer. Overall, both the different fragmentation probability of Ag(I) 2,2-dimethylbutanoate and Ag(I) benzoate and the different reactivity of the radical species released upon electron impact rationalise the ESD and RAIRS results (Section 3.2) and support that aliphatic carboxylate ligands are superior ligands for FEBID precursors.

## 5. Conclusions

We investigated the electron-induced decomposition of Ag(I) 2,2-dimethylbutanoate, a precursor that yields deposits with high Ag contents in FEBID [7] and compared its reactivity under irradiation to other Ag(I) carboxylates with different hydrocarbon structures attached to the carboxylate group. In addition, the fragmentation behaviour of Ag(I) 3,3-dimethyl-1-butynyl was studied. ESD and RAIRS experiments performed on sublimates on an inert surface in combination with calculations of the average local ionization energy (ALIE) of the different Ag(I) compounds yield a comprehensive picture of the factors that determine the efficiency of ligand removal as result of electron irradiation. In particular, carboxylates with saturated hydrocarbon structure are decomposed with high efficiency to yield CO_2_ and an alkene that derives from the hydrocarbon group by loss of atomic hydrogen. In contrast, loss of hydrogen from a phenyl radical that is released upon electron-induced expulsion of CO_2_ from the ligand of Ag(I) benzoate is not favourable because it would result in a highly strained ring structure with triple bond. Aromatic radicals therefore preferably react by cross-linking with other fragments and thus retain carbon in the nonvolatile deposit formed under electron exposure.

Another factor that determines the electron-induced fragmentation of the Ag(I) compounds is the site within in the molecule from which an electron is removed upon ionization. As shown by the calculations, the ALIE is lowest on the carboxylate group in the case of Ag(I) 2,2-dimethylbutanoate. Ionization thus preferably destabilises the carboxylate group and triggers expulsion of CO_2_. In contrast, CC double or triple bonds, such as present in Ag(I) benzoate and Ag(I) 3,3-dimethyl-1-butynyl, represent alternative sites within the molecule where electron density can be easily removed. Loss of an electron from multiple CC bonds, however, does not favour dissociation. This rationalizes the slow decomposition of Ag(I) benzoate under electron exposure as obvious from the present RAIRS results and provides an explanation for the high stability of Ag(I) 3,3-dimethyl-1-butynyl, as noted previously. As a general guideline for the design of novel FEBID precursors, our results thus indicate that aliphatic carboxylate ligands are a preferential choice because (i) ionization upon electron impact in fact removes charge preferably from the carboxylate group and (ii) this triggers decomposition to stable volatile products.

## Data Availability

Data presented in this study are available on request from the corresponding author.

## Supplementary Materials

The following supporting information can be downloaded at: https://www.mdpi.com/article/10.3390/nano12101687/s1, Figure S1: Full range infrared spectra of Ag(I) 2,2-dimethylbutanoate and Ag(I) benzoate. Table S1: Vibrational band positions and assignments for Ag(I) 2,2-dimethylbutanoate [21,40,41,42,43]. Table S2: Vibrational band positions and assignments for Ag(I) benzoate [37,38,39,40,41,42,43]. Table S3: Vibrational band positions and assignments for Ag(I) 3,3-dimethyl-1-butynyl [45,46]. Figure S2: ATR spectra and RAIR spectra of sublimates of Ag(I) 2,2-dimethylbutanoate, Ag(I) benzoate, and Ag(I) 3,3-dimethyl-1-butynyl used in the different experiments. Table S4: Comparison of RAIRS and ATR intensities for average sublimate thickness estimation [26]. Figure S3: Optical microscopy of sublimate layers of Ag(I) 2,2-dimethylbutanoate, Ag(I) benzoate, and Ag(I) 3,3-dimethyl-1-butynyl. Table S5: XPS Au signals used for an estimate of the sublimate thickness. Figure S4: Simulated relative attenuation of XPS Au4d and Au4f signals for sublimate layers [50]. Figure S5: RAIRS of Ag(I) 2,2-dimethylbutanoate before and after the electron exposures. Figure S6: RAIRS and ESD of sublimates with increasing amount of Ag(I) 2,2-dimethylbutanoate. Figure S7: ESD mass spectra recorded from sublimates of Ag(I) 2,2-dimethylbutanoate and mass spectra of CO_2_, H_2_O, and 2-methyl-2-butene. Figure S8: ESD mass spectra to exclude additional H_2_ production from cl-BPT SAM during irradiation. Figure S9: ESD mass spectra recorded from sublimates of Ag(I) 2,2-dimethylbutanoate and perdeuterated Ag(I) 2,2-dimethylbutanoate. Table S6: ESD signals and assignments for Ag(I) 2,2-dimethylbutanoate and perdeuterated Ag(I) 2,2-dimethylbutanoate. Figure S10: ESD mass spectra recorded from sublimates of Ag(I) 2,2-dimethylbutanoate, Ag(I) 2-methylpentanoate, Ag(I) hexanoate, and Ag(I) heptanoate. Figure S11: RAIR spectra of Ag(I) 2,2-dimethylbutanoate, Ag(I) 2-methylpentanoate, Ag(I) hexanoate, and Ag(I) heptanoate before and after electron irradiation. Figure S12: RAIR spectra of Ag(I) 3,3-dimethyl-1-butynyl before and after irradiation. Figure S13: ESD mass spectra recorded from a thick sublimate layer of Ag(I) 3,3-dimethyl-1-butynyl. Figure S14: ESD kinetic experiment of Ag(I) 2,2-dimethylbutanoate. Figure S15: RAIRS of Ag(I) 2,2-dimethylbutanoate recorded before and after different electron exposures. References in Supplementary Materials [14,25,49,51].
