# Supplementary material for: Electron-Induced Decomposition of Different Silver(I) Complexes: Implications for the Design of Precursors for Focused Electron Beam Induced Deposition
Source: Nanomaterials (Basel). 2022 May 15;12(10):1687. doi: 10.3390/nano12101687 (PMC9147827; doi:10.3390/nano12101687)
Supplement: Supplementary file 1 [file nanomaterials-12-01687-s001.zip › nanomaterials-1691911-supplementary.pdf]

Supporting Information

Electron-Induced Decomposition of Different Silver(I) Complexes: Implications for the Design of Precursors for Focused Electron Beam Induced Deposition

Petra Martinović [1], Markus Rohdenburg [1,2], Aleksandra Butrymowicz [3], Selma Sarigül [1], Paula Huth [2], Reinhard Denecke [2], Iwona B. Szymańska [3] and Petra Swiderek [1,*]

[1] Institute for Applied and Physical Chemistry (IAPC), Fachbereich 2 (Chemie/Biologie), University of Bremen, Leobener Str. 5 (NW2), 28359 Bremen, Germany; petra@uni-bremen.de (P.M.); markus.rohdenburg@uni-leipzig.de (M.R.); selma.sariguel@studium.uni-hamburg.de (S.S.)

[2] Wilhelm-Ostwald-Institute for Physical and Theoretical Chemistry (WOI), Leipzig University, Linnéstr. 2, 04103 Leipzig, Germany; che09dup@studserv.uni-leipzig.de (P.H.); denecke@uni-leipzig.de (R.D.)

[3] Faculty of Chemistry, Nicolaus Copernicus University in Toruń, Gagarina 7, 87-100 Toruń, Poland;
aleksandra.butrymowicz@doktorant.umk.pl (A.B.); pola@umk.pl (I.B.S.)

* Correspondence: swiderek@uni-bremen.de

1. Infrared spectra of Ag 2,2-dimethylbutanoate and Ag benzoate

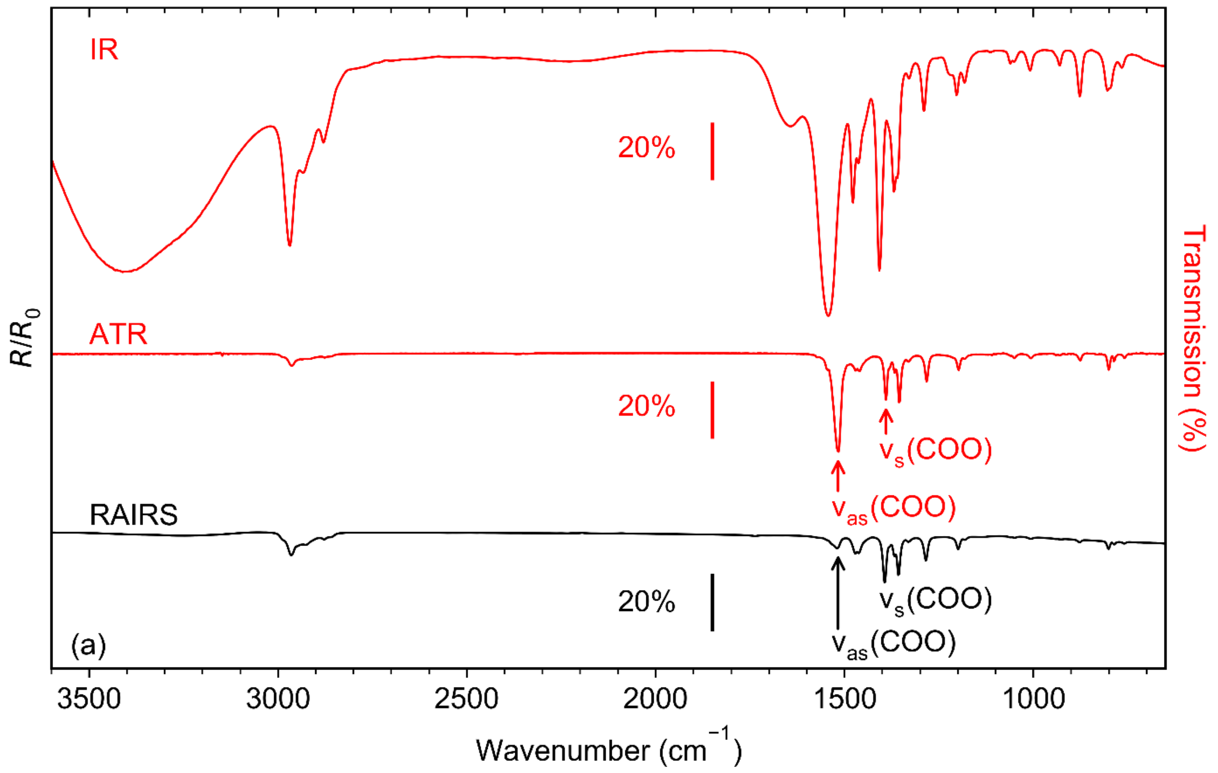

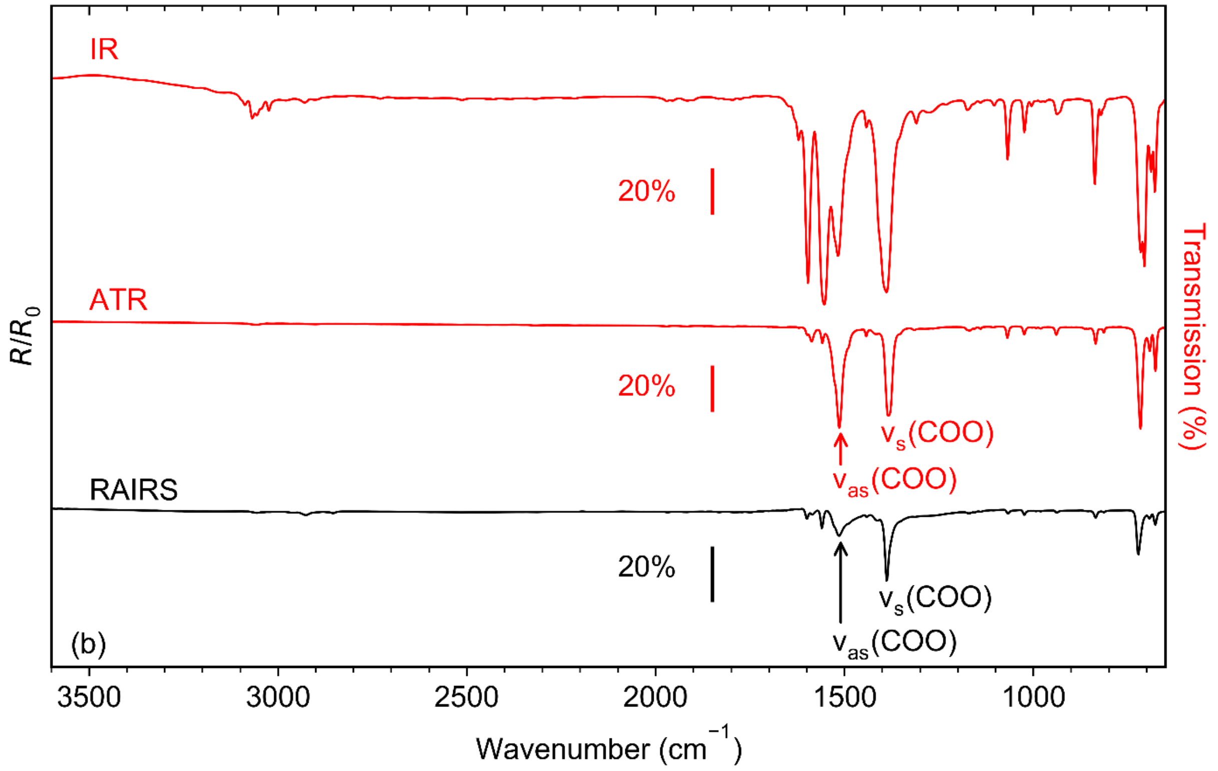

Figure S1. Infrared spectra of (a) Ag(I) 2,2-dimethylbutanoate and (b) Ag(I) benzoate. The two upper spectra in each frame were recorded in transmission from KBr pellet (IR) and by attenuated total reflectance (ATR) from the as-synthesized compound. The bottom spectrum (RAIRS) was recorded in reflection from the compounds sublimated onto cl-BPT/Au substrates. The data are the same as shown for a smaller range of wavenumbers in Figure 2 of the main manuscript.

Table S1. Vibrational band positions and assignments for Ag(I) 2,2-dimethylbutanoate.

Ag(I) 2,2-dimethylbutanoate				
**Assignment[a]**	**bulk IR (cm^{-1})**	**bulk ATR (cm^{-1})**	**RAIRS (cm^{-1})**	**bulk IR (cm^{-1})[b]**
N(CH)	2969<br>2934<br>2880	2962<br>2919<br>2875	2963<br>2929<br>2877	
$v_{as}(COO^-)$ of monodentate coordination[c]	1544	1547		1548
$v_{as}(COO^-)$ of bridging coordination[c]		1515	1520	
$\delta_{as}(CH_3)$, $\delta(CH_2)$	1477<br>1464	1471	1471	1477
$v_s(COO^-)$	1408	1389	1394	1405
$\delta_s(CH_3)$	1369<br>1359	1356	1357	1366<br>1357
$\delta_w(CH_2)$, $\delta_t(CH_2)$	1290	1282	1285	1290
Skeletal vibrations	1220<br>1204<br>1183<br>1061<br>1050<br>1008<br>930<br>877<br>800	1198<br>1182<br>1061<br>1051<br>1007<br>930<br>876<br>800	1200<br>1182<br>1062<br>1050<br>1008<br>928<br>877<br>800	

a v = stretching, δ = deformation, δ_r = rocking, δ_w = wagging, δ_t = twisting.
b from KBr pellet [1].
c see Ref. [2–5].

Table S2. Vibrational band positions and assignments for Ag(I) benzoate.

Ag(I) benzoate				
Assignment[a,b]	bulk IR (cm^{-1})	bulk ATR (cm^{-1})	RAIRS (cm^{-1})	bulk IR (cm^{-1})[b]
ν(CH)	3088, 3068, 3056, 3024, 2930	3050, 2900	3060, 2928, 2855	3050 vw
	1622	1622		
ν(CC)	1598	1599	1602	1596 vs
$\nu_{as}(COO^-)$ of monodentate coordination[c]	1553	1559	1562	1553 vs<br>1560[d]
$\nu_{as}(COO^-)$ of bridging coordination[c]	1517	1514	1516	1518[d]
ν(CC)	1442	1418	1415	1407-1447 s-w[e]
ν_s(COO)	1390	1384	1390	1412 s<br>1397 sh
ν(CC)	1310	1315	1314	1306 vw
β(CH)	1069	1069	1068	1067 w
β(CH)	1025	1025	1024	1023 vw
$\beta_s(COO^-)$	838	835	837	842 w
γ(CH)	820	814	813	817 vw
$\gamma_s(COO^-)$	716	716	724	711 s
ϕ(CC)	679	676	681	683 sh

a Nomenclature is ν = stretching, β = in plane deformation / angle deformation, γ = out of plane deformation, ϕ = out of plane ring deformation. Note that assignments for last four entries are not unique, see also Ref. [6].
b from KBr pellet and Nujol. [7].
c see Ref. [2–5].
d values for Ag stearate from Ref. [3].
e values for different alkali benzoates, see Ref. [8].

Table S3. Vibrational band positions and assignments for Ag(I) 3,3-dimethyl-1-butynyl.

Ag(I) 3,3-dimethyl-1-butynyl				
**Assignment[a,b]**	**bulk IR (cm^{-1})**	**bulk ATR (cm^{-1})**	**RAIRS (cm^{-1})**	**liquid IR[b]**
v(≡CH)	-	-	-	3309 s
$v(CH_3)$	2966<br>2947<br>2920<br>2895<br>2863	2966<br>2944<br>2918<br>2893<br>2860	2967<br>2947<br>2920<br>2894<br>2863	2971 s<br>2954 sh<br>2935 vw<br>2906 m<br>2872 s
v(C≡C)	2056	2053	2056	2106 m
$v_{as}(COO^-)$ of monodentate coordination[c]	1551	1548	1550	
v_s(COO)[c]	1393	1389	1390	
$\delta(CH_3)$	1472<br>1453<br>1361	1471<br>1452<br>1359	1473<br>1453<br>1361	1476 m<br>1456 m<br>1367 s
$\delta_r(CH_3)$ + v(CC)	1244	1242	1245	1248 s
v(CC) + $\delta(CH_3)$	1203	1202	1204	1204 m
$v(CC_3)$	884	895	894	882 m

a v = stretching, δ = deformation, δ_r = rocking

b data for tert-butylacetylene from Ref. [9], compare also Ref. [10] for assignments.

c Bands resulting from formation of carboxylate complex by insertion of CO_2 into the Ag-C bond.

2. Estimate of the thickness of sublimated samples

All sublimated samples were inspected by reflection absorption infrared spectroscopy (RAIRS) prior to electron irradiation. Infrared spectra obtained by attenuated total reflectance (ATR-IR) measurements were used as reference by which we deduce, from the RAIRS data, information on how the absolute amounts of material present in the sublimates of Ag(I) 2,2-dimethylbutanoate, Ag(I) benzoate, and Ag(I) 3,3-dimethyl-1-butynyl compare to each other. Some samples were, in addition, investigated by optical microscopy and X-ray photoelectron spectroscopy (XPS) to obtain more insight into the distribution of the material on the surface. We explain here the arguments on which the conclusions presented in the main manuscript are based.

The RAIRS data for all samples that were used in the experiments presented in the main text are shown in Figure S2, together with the ATR-IR spectra of Ag(I) 2,2-dimethylbutanoate, Ag(I) benzoate, and Ag(I) 3,3-dimethyl-1-butynyl. The infrared intensities were evaluated to obtain a rough estimate of the relative average thickness of the sublimate layers. This analysis is based on a comparison of ATR-IR and RAIRS intensities for selected bands. The intensities are measured as the peak height because the width of the bands in the different spectra is comparable. To support this procedure, we argue that the penetration depth of the evanescent IR wave into the samples pressed onto the ATR crystal should be similar for all three compounds. We use the ATR-IR intensities of bands with similar wavenumber as reference (see Table S4) to avoid wavenumber-dependent distortion of the spectra [11] and have checked by gradually increasing the force applied by the ATR piston that the intensities were in saturation. Furthermore, and in line with previous results (see for instance Refs. [12–14]), our estimate assumes that, for sufficiently thin molecular layers, the RAIRS intensities increase linearly with the amount of material as long as the average orientation of the molecules remains the same. Such orientation effects are absent from the present RAIRS data which show, for each compound, a generally constant pattern (Figure S2). The obvious intensity fluctuations between different samples of the same compound included in Figure S2 thus reflect variations in the amount of material present in the samples.

For each of the three Ag(I) compounds, the band intensities in RAIRS summarized in Table S4 are compared to those found in the ATR-IR spectra. Most importantly, the ratio between the RAIRS intensity of the sample used in the ESD experiment (Figures 5 and 6 of main manuscript) and the ATR-IR intensity is higher for Ag(I) 2,2-dimethylbutanoate than for the other two compounds. This indicates that the average thickness of Ag(I) benzoate and Ag(I) 3,3-dimethyl-1-butynyl sublimate layers does not exceed that of Ag(I) 2,2-dimethylbutanoate. Based on this result, the decomposition efficiency of the three compounds can be safely compared as described in Section 3.2 of the main manuscript.

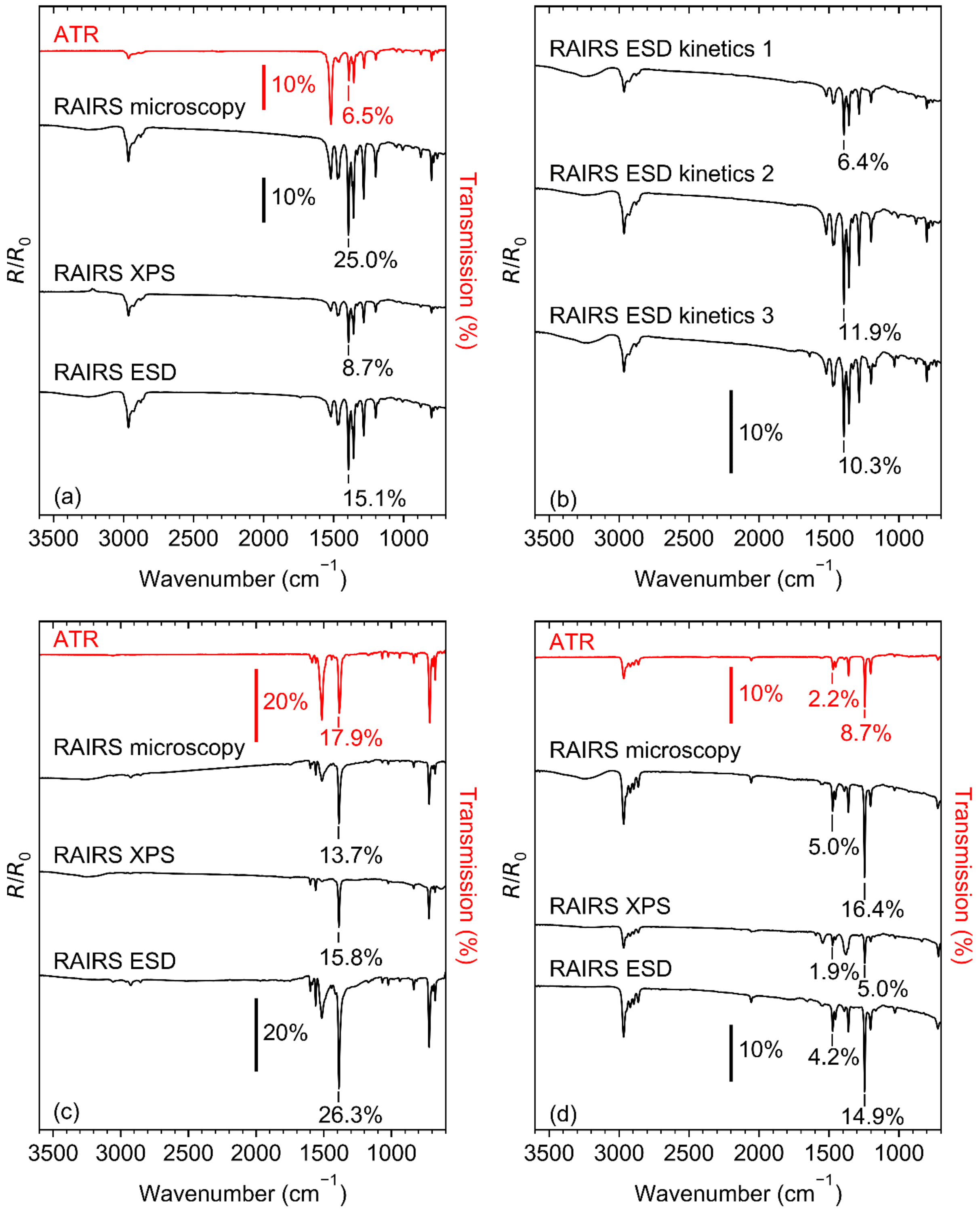

Figure S2. ATR spectra (red) and RAIR spectra (black) of sublimates of (a,b) Ag(I) 2,2-dimethylbutanoate, (c) Ag(I) benzoate, and (d) Ag(I) 3,3-dimethyl-1-butynyl used in the different experiments presented in this work. The labels indicate in which experiments the RAIRS samples were subsequently used. See Table S4 for details.

Table S4. RAIRS intensities of selected bands for samples used in the experiments described in the main text and corresponding ATR intensities given in % as used as reference for an estimate of the average sublimate thickness. Values in parentheses establish a comparison between intensities in RAIRS and ATR with the latter being set to 100 for convenience. It is assumed that ATR intensities reflect the same amount of material for all three compounds.

	**Ag(I) 2,2-dimethyl-butanoate**	**Ag(I) benzoate**	**Ag(I) 3,3-dimethyl-1-butynyl**
Selected band[a]	$\nu_s(COO^-)$ 1390 cm^{-1}	$\nu_s(COO^-)$ 1390 cm^{-1}	$\delta(CH_3)$ 1360 cm^{-1}
ATR[b]	6.5% (100)	17.9% (100)	8.7% (100)
RAIRS microscopy (Figure S3)	25.0% (385)	13.7% (77)	16.4% (189)
RAIRS ESD (Figures 5 and 6)	15.1% (232)	26.3% (145)	14.9% (171)
RAIRS XPS (Figure S2)	8.7% (134)	15.8% (88)	5.0% (57)
RAIRS ESD kinetics 1 (Figure 9)	6.4% (98)		
RAIRS ESD kinetics 2 (Figure 10a)	11.9% (183)		
RAIRS ESD kinetics 3 (Figure 10b)	10.3% (158)		

a The analysis is based on vibrational bands with similar wavenumbers to avoid effects of wavenumber dependence of the ATR data. See Ref. [11].

Optical microscopy (Figure S3) shows that the sublimate layers of Ag(I) 2,2-dimethylbutanoate and Ag(I) benzoate exhibit larger agglomerated crystallites while the sublimate of Ag(I) 3,3-dimethyl-1-butynyl is more homogeneous. We note that according to the RAIRS intensities (Table S4), the sample of Ag(I) 2,2-dimethylbutanoate used in microscopy was thicker than samples used in electron irradiation experiments. However, even this amount of material is thin enough to warrant that the orientation of the molecules in the sublimate is governed by the surface (see also Section 3.1 of main manuscript). In contrast, the RAIR spectrum of a sublimate that has been prepared from a larger amount of material (see RAIRS denoted 5 mg in Figure S6) has a significantly higher intensity and closely matches the ATR-IR spectrum indicating that the orientation of the molecules is no longer governed by the surface. This effect most likely indicates that larger crystallites begin to grow independently of the underlying surface and dominate the RAIR spectrum. This suggest that the agglomerated crystallites seen in Figure S3a are not dominant in RAIRS. As the optical microscopy image does not allow to conclude on the presence of a thin layer between the crystals, XPS was used as a further tool to characterize the sample.

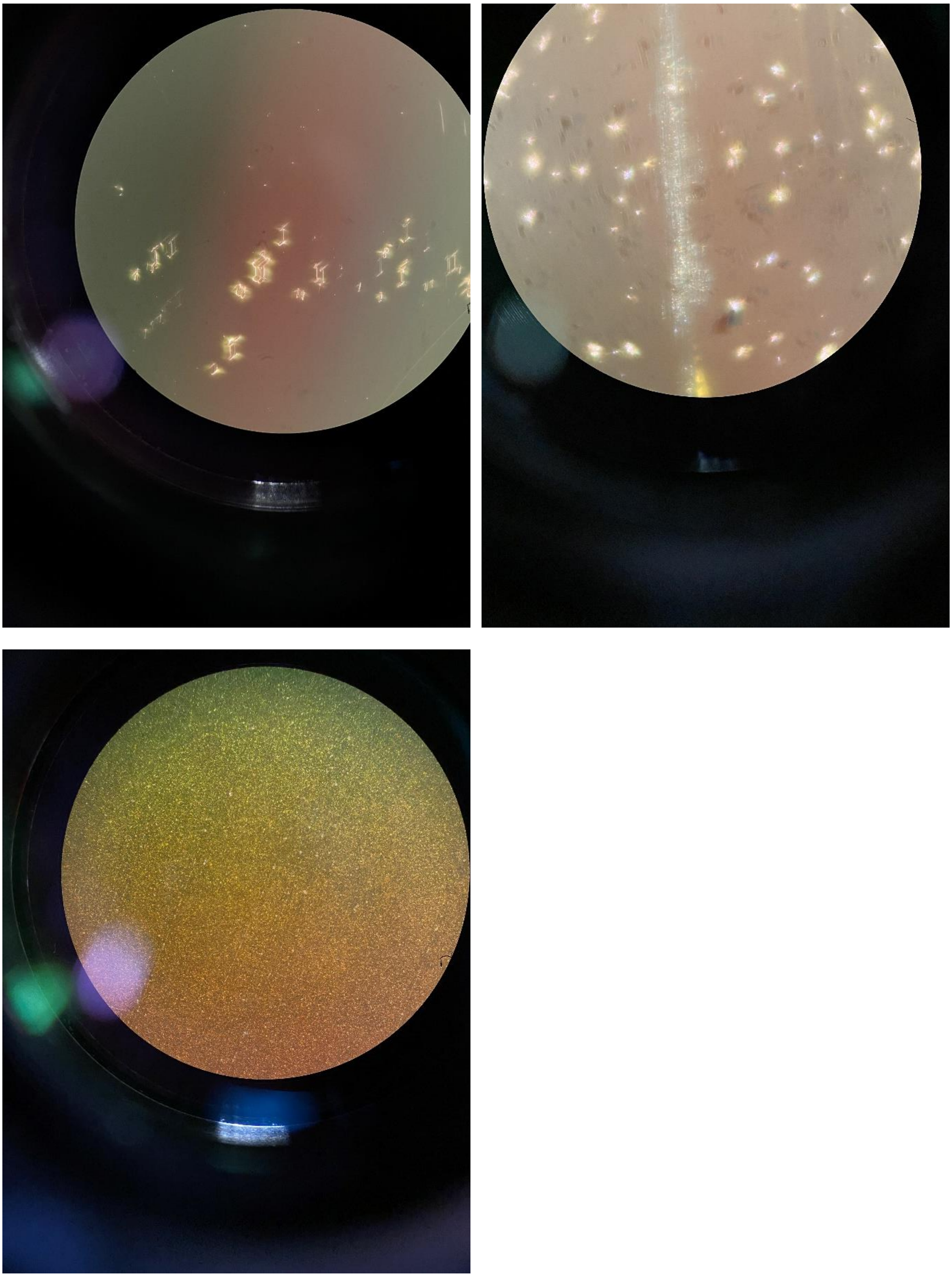

Figure S3. Optical microscopy (40x) of sublimate layers of (a) Ag(I) 2,2-dimethylbutanoate, (b) Ag(I) benzoate, and (c) Ag(I) 3,3-dimethyl-1-butynyl prepared on Au substrates without cl-BPT SAM. The circular view field has a physical diameter of 0.5 mm.

XPS has been performed to obtain more information on the surface coverage of the sublimate layers. To this end, the Ag(I) complexes were sublimated on Au substrates without cl-BPT SAM and the attenuation of signals for electron emission from Au4d and Au4f levels from the underlying substrate, which possess different kinetic energies, was evaluated using the software UNIFIT 2021 [15]. This analysis assumes a uniform thickness which is calculated from the ratio of the integrated intensities of the two signals after correction for the respective ionization cross sections and the spectrometer transmission function taking account of the different electron effective attenuation lengths of the Au4d and Au4f photoelectrons (See equation (10) in Ref. [15]). Values for the attenuation length of 33 Å for Au4d photoelectrons and 39 Å for Au4f photoelectrons were obtained for Ag(I) 2,2-dimethylbutanoate (density of 1.15 g/cm^3) using the NIST Electron Effective-Attenuation-Length Database [16]. The same values were used for the other two compounds where densities have not been reported. The thicknesses derived from these values under the assumption of a homogeneous surface coverage are summarized in Table S5.

Table S5. XPS Au signals used for an estimate of the sublimate thickness.

	**Ag(I) 2,2-dimethyl-butanoate**	**Ag(I) benzoate**	**Ag(I) 3,3-dimethyl-1-butynyl**
Area Au 4d	36	48	44
Area Au 4f	63	52	54
Ratio Au4d/Au4f	0.57	0.92	0.82
Thickness according to equation (12) [15]	12 nm	1.7 nm	4.4 nm
Conclusion from models shown in Figure S4	Majority of the substrate must be covered by sublimate	Up to 1/3 of surface may be void of sublimate	Up to 10% of the substrate may be void of sublimate

According to optical microscopy (Figure S3), the assumption of a homogeneous thickness is questionable in the case of the sublimate layers considered here. Therefore, we discuss here the effect of partial surface coverage on the intensity ratio of the Au4d and Au4f signals using a simple model. In the simplest case, the model describes a surface covered by an amount of material that, when homogeneously distributed, results in a thickness *d*. We then consider situations where the same amount of material covers only a fraction A of the surface and the material is stacked again with homogeneous thickness d/A within this area. The intensity of a photoelectron signal that stems with initial intensity I_0 from the underlying Au surface then results as

$$I = I_0 \cdot (1 - A) + I_0 \cdot A \cdot e^{-d/A\lambda}.$$

Here, λ is the electron effective attenuation length for a given photoelectron kinetic energy. Using this equation and the values for the attenuation length given above, the resulting intensities for Au4d and Au4f were calculated and their ratio is plotted as function of surface coverage A in Figure S4a (enlargement thereof in Figure S4b) for a series of thicknesses *d*.

Note that for a value of $A = 1$ and the thicknesses listed in Table S5, the plots would in fact reproduce the experimental intensity ratios. A modified version of this model (Figure S4c and enlargement thereof in Figure S4d) reflects a certain roughness. Again, the model represents an amount of material that, when homogeneously distributed, results in a thickness *d* and the material is simply redistributed when describing partial coverage. However, in this case the thickness in 50% of the covered area is reduced by 1/3 while it is increased by the same amount in the remaining 50%. In this case, the intensity of a given photoelectron signal as compared to a bare Au surface would result as

$$I = I_0 \cdot (1 - A) + 0.5 \cdot I_0 \cdot A \cdot e^{-2d/3A\lambda} + 0.5 \cdot I_0 \cdot A \cdot e^{-4d/3A\lambda}.$$

Both models yield qualitatively similar results. In particular, Figure S4 shows that an intensity ratio of 0.57 as observed for Ag(I) dimethylbutanoate is a strong indication that the Au substrate is in fact predominantly covered by the sublimate layer. Considering that the sublimate sample of Ag(I) dimethylbutanoate used for XPS was significantly thinner than the one imaged by optical microscopy (see RAIRS data in Table S4), we conclude that the crystallites seen in Figure S3a most likely have grown on top of a more homogeneous layer that covered the entire substrate. The result is less clear for Ag(I) benzoate and Ag(I) 3,3-dimethyl-1-butynyl. According to Figure S4, the intensity ratio of 0.82 obtained for the latter would also be consistent with a layer that is on average thicker than 4.4 nm but leaves a minor part of the substrate (<10%) uncovered. An even larger part of the surface can be void of sublimate in the case of Ag(I) benzoate. In this case the amount of sublimate would even more exceed that of a homogeneous 1.7 nm layer. However, the intensity ratios as function of fraction of covered surface obtained from the two models suggest that at least 66% of the surface would even need to be covered by sublimate of Ag(I) benzoate to reproduce the experimental ratio of 0.92. The infrared results (Table S4) suggest that the amount of material present in the sublimate of Ag(I) benzoate used for XPS is about 65% of the amount of Ag(I) dimethylbutanoate used for XPS.Taken together, this would translate to an average thickness of the of Ag(I) benzoate sublimate that is unlikely to exceed that of Ag(I) dimethylbutanoate. An analogous estimate for Ag(I) 3,3-dimethyl-1-butynyl arrives at the same conclusion. Based on these arguments, we can safely conclude that the amount of material present in the sublimates of Ag(I) benzoate and Ag(I) 3,3-dimethyl-1-butynyl generally did not exceed the amount of material present in sublimates of Ag(I) dimethylbutanoate. This conclusion is important for the evaluation of the results presented in Section 3.2 of the main manuscript.

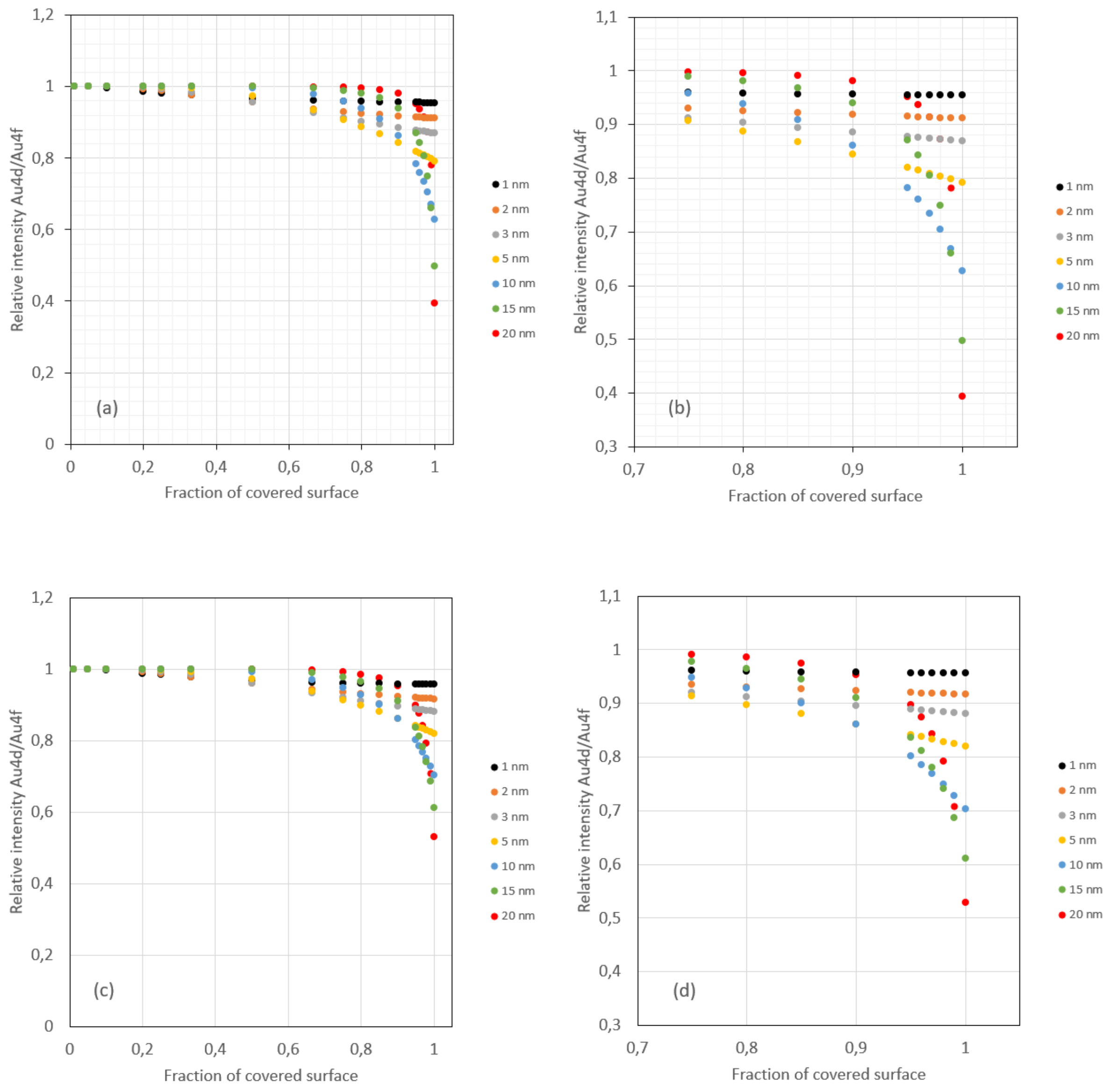

Figure S4. Simulated relative attenuation of XPS Au4d and Au4f signals for sublimate layers produced from different amounts of material stated as homogeneous thickness for a fully covered surface in the plot. For partial coverage the same amount of material is either contained with homogeneous thickness within the covered areas (a,b) or its thickness is reduced by 1/3 in 50% of the covered area while it is increased by the same amount in the remaining 50% (c,d). See text for further details of the model.

3. Electron-induced decomposition of Ag(I) 2,2-dimethylbutanoate

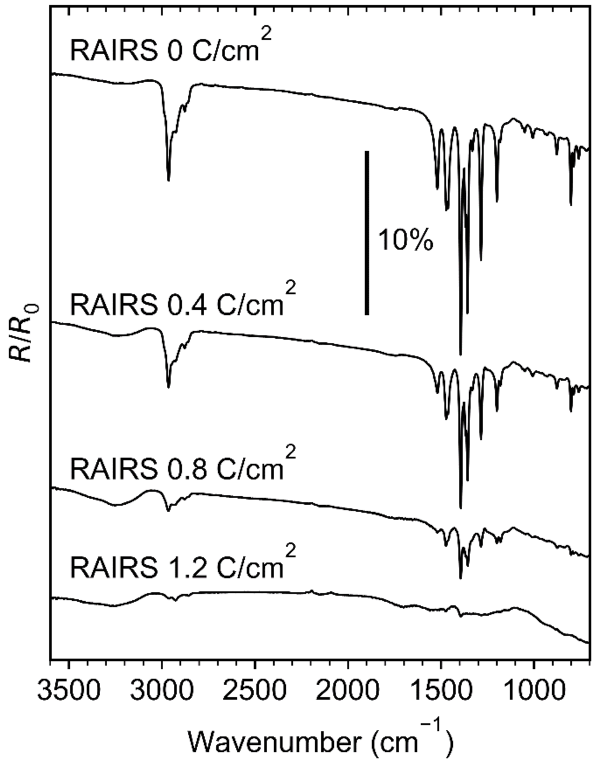

Figure S5. RAIRS of Ag(I) 2,2-dimethylbutanoate recorded before (0 C/cm^2) and after the electron exposures stated in the graph at an electron energy of 50 eV.

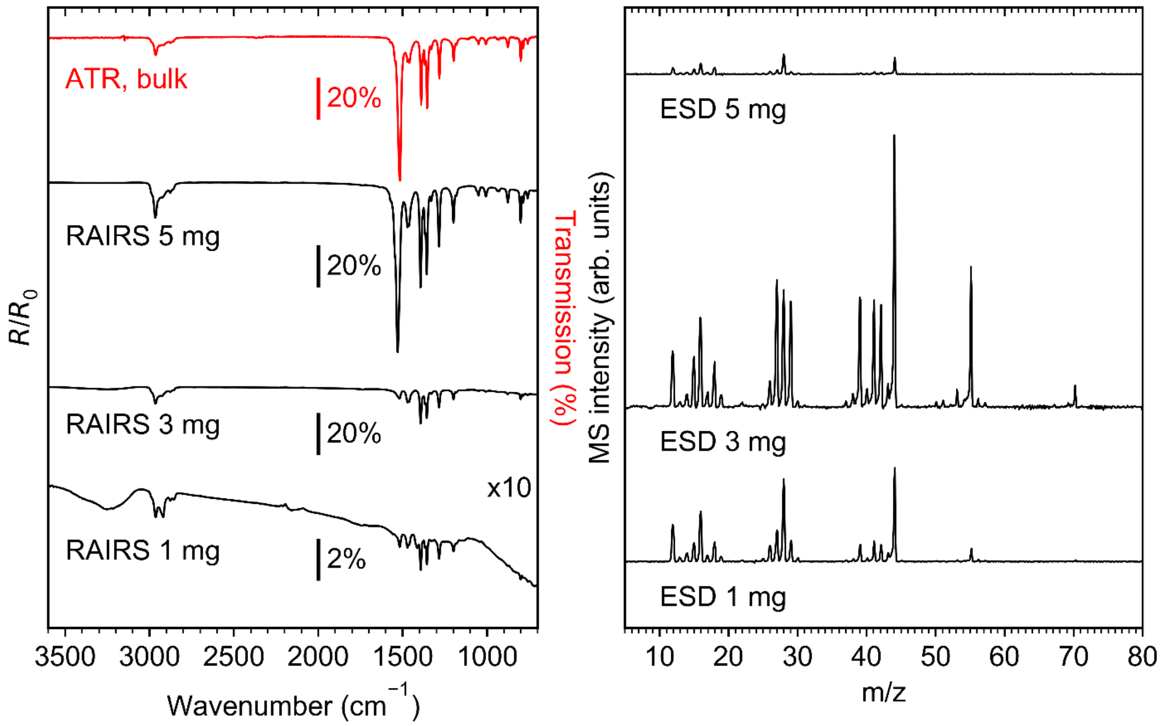

Figure S6. RAIRS of sublimates prepared from increasing amounts of Ag(I) 2,2-dimethylbutanoate as indicated in the graph and comparison to ATR-IR of the same compound (left) as well as ESD mass spectra obtained from these samples upon electron irradiation at 50 eV.

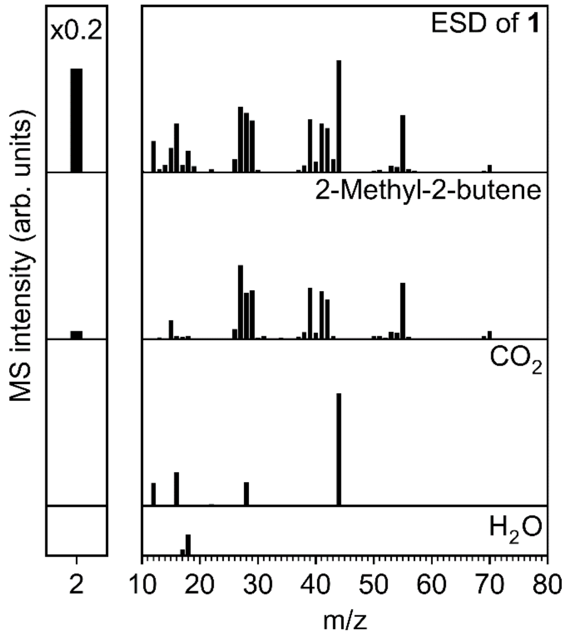

Figure S7. Mass spectrum (m/z 0-80) of the volatile species produced upon ESD ($E_0 = 50$ eV) from Ag(I) 2,2-dimethylbutanoate and mass spectra obtained using the same QMS upon leaking of CO_2, H_2O, and 2-methyl-2-butene into the vacuum chamber.

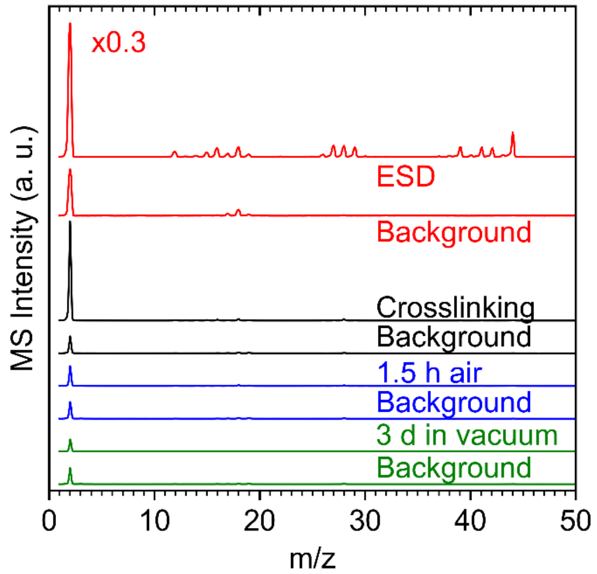

Figure S8. Red: ESD mass spectrum recorded during electron irradiation ($E_0 = 50$ eV) of Ag(I) 2,2-dimethylbutanoate. Black: ESD mass spectrum ($E_0 = 500$ eV) recorded during electron-induced crosslinking of a BPT-SAM. Blue: ESD mass spectrum ($E_0 = 500$ eV) recorded from the same cl-BPT-SAM after 1.5 h exposure to ambient conditions. Green: ESD mass spectrum ($E_0 = 500$ eV) recorded from the same cl-BPT-SAM after 3 days storing in vacuum. Note that the spectra represent raw data that have not been corrected by subtracting the background spectra recorded before the start of the respective irradiation experiment and included here as bottom curves of each data. The result shows that electron irradiation of a cl-BPT SAM does not lead to production of H_2 above the level present in the vacuum chamber as residual gas.

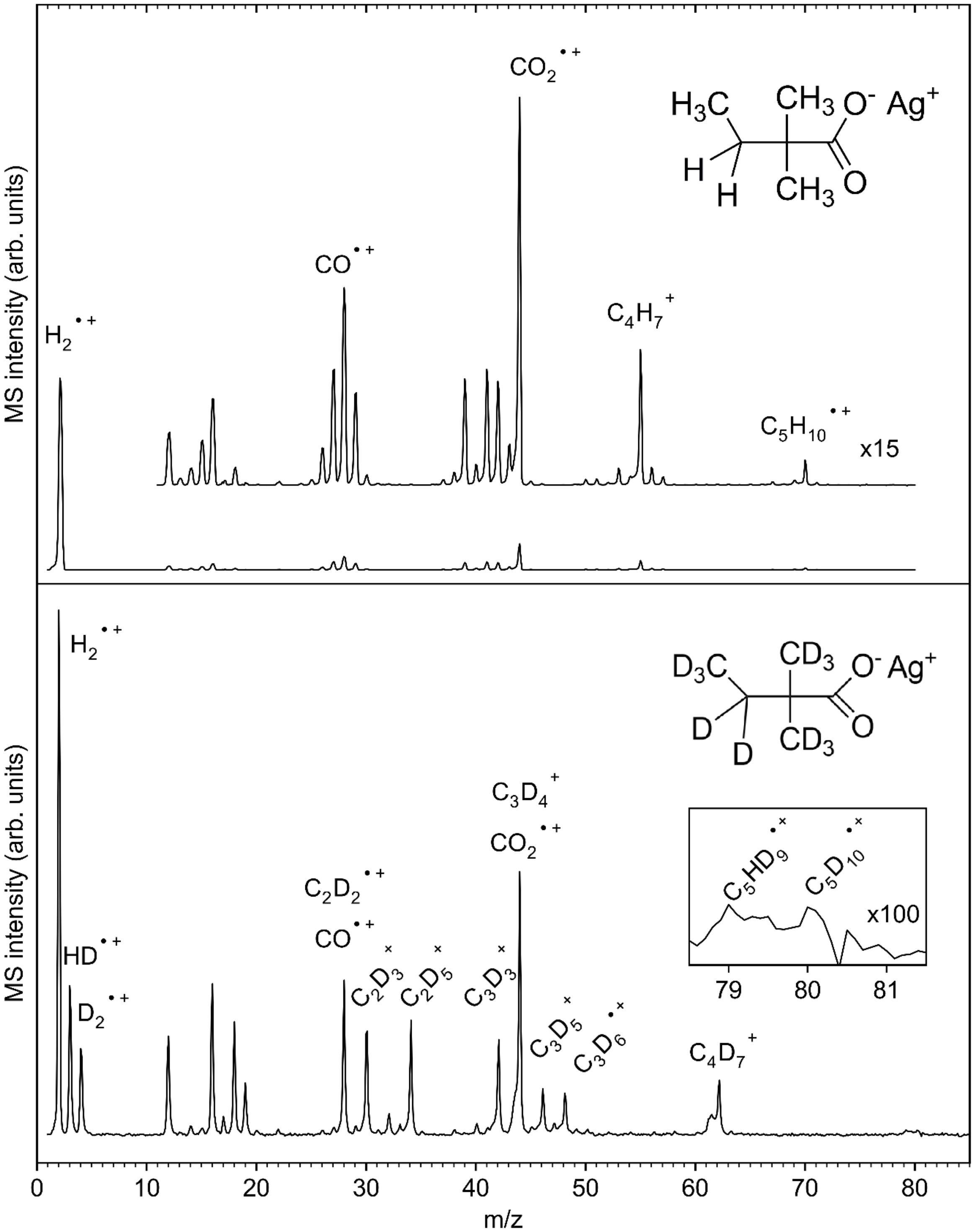

Figure S9. ESD mass spectra recorded from sublimates of Ag(I) 2,2-dimethylbutanoate (top) and perdeuterated Ag(I) 2,2-dimethylbutanoate (bottom) upon electron irradiation at 100 eV. The higher energy was used to obtain higher ESD intensities. The signals are also listed in Table S6.

Table S6. ESD signals and assignments for Ag(I) 2,2-dimethylbutanoate and perdeuterated Ag(I) 2,2-dimethylbutanoate as seen in Figure S9.

**Ag 2,2-dimethylbutanoate**		**Perdeuterated Ag 2,2-dimethylbutanoate**			
**Fragment**	**m/z**	**Fragment**	**m/z**	**H exchanged fragment**	**m/z**
$[C_5H_{10}]^{\bullet+}$	70	$[C_5D_{10}]^{\bullet+}$	80	$[C_5HD_9]^{\bullet+}$	79
$C_4H_7^+$	55	$C_4D_7^+$	62	$C_4HD_6^+$	61
$[CO_2]^{\bullet+}$	44				
$[C_3H_6]^{\bullet+}$	42	$[C_3D_6]^{\bullet+}$	48		
$C_2H_5^+$	41	$C_3D_5^+$	46		
$C_3H_4^+$	40 weak	$[CO_2]^{\bullet+}$, $C_3D_4^+$	44		
$C_3H_3^+$	39	$C_3D_3^+$	42		
		$C_3D_2^+$	40		
$C_2H_5^+$	29	$C_2D_5^+$	34		
$CO^{\bullet+}$, $[C_2H_4]^{\bullet+}$	28	$[C_2D_4]^{\bullet+}$	32 weak		
$C_2H_3^+$	27	$C_2D_3^+$	30		
$[C_2H_2]^{\bullet+}$	26	$CO^{\bullet+}$, $[C_2D_2]^{\bullet+}$	28		
$[H_2O]^{\bullet+}$	18				
$O^{\bullet+}$, $[CH_4]^{\bullet+}$	16	$[CD_4]^{\bullet+}$	20 weak	$[CHD_3]^{\bullet+}$	19
CH_3^+	15	$[H_2O]^{\bullet+}$, CD_3^+	18		
$CH_2^{\bullet+}$	14	$O^{\bullet+}$, $CD_2^{\bullet+}$	16		
$C^{\bullet+}$	12	$C^{\bullet+}$	12		
		$[D_2]^{\bullet+}$	4		
		$[HD]^{\bullet+}$	3		
$[H_2]^{\bullet+}$	2	$[H_2]^{\bullet+}$	2		

4. Electron-induced degradation of Ag carboxylates with different alkyl side chain

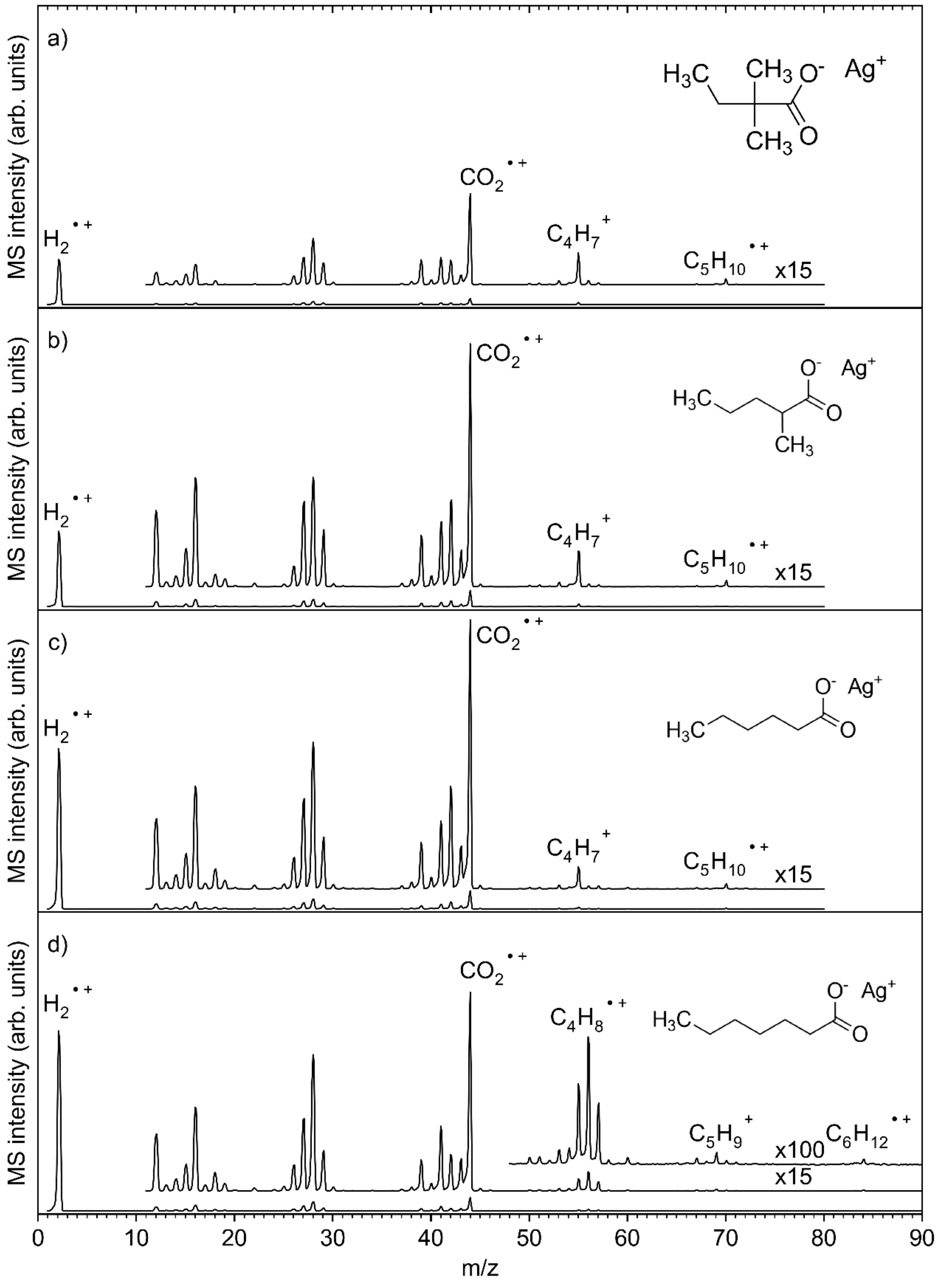

Figure S10. ESD mass spectra of the volatile species produced from sublimates of (a) Ag(I) 2,2-dimethylbutanoate, (b) Ag(I) 2-methylpentanoate, (c) Ag(I) hexanoate, and (d) Ag(I) heptanoate upon electron irradiation at 100 eV. The higher energy was used to obtain higher ESD intensities.

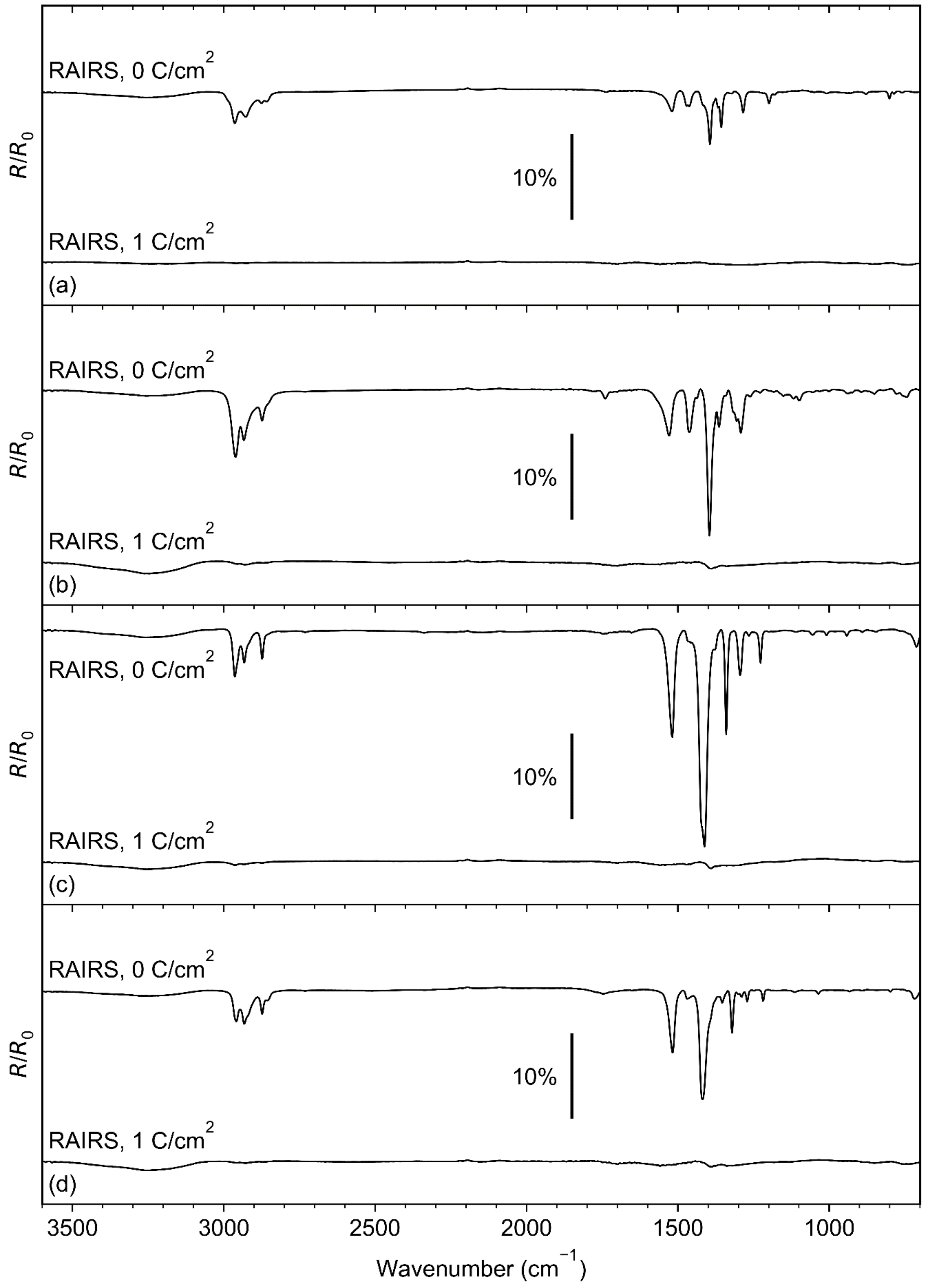

Figure S11. RAIR spectra of (a) Ag(I) 2,2-dimethylbutanoate, (b) Ag(I) 2-methylpentanoate, (c) Ag(I) hexanoate, and (d) Ag(I) heptanoate before (0 C/cm^2) and after electron irradiation (1 C/cm^2) at E_0 = 100 eV.

5. Electron-induced degradation of a thick Ag(I) 3,3-dimethyl-1-butynyl sublimate

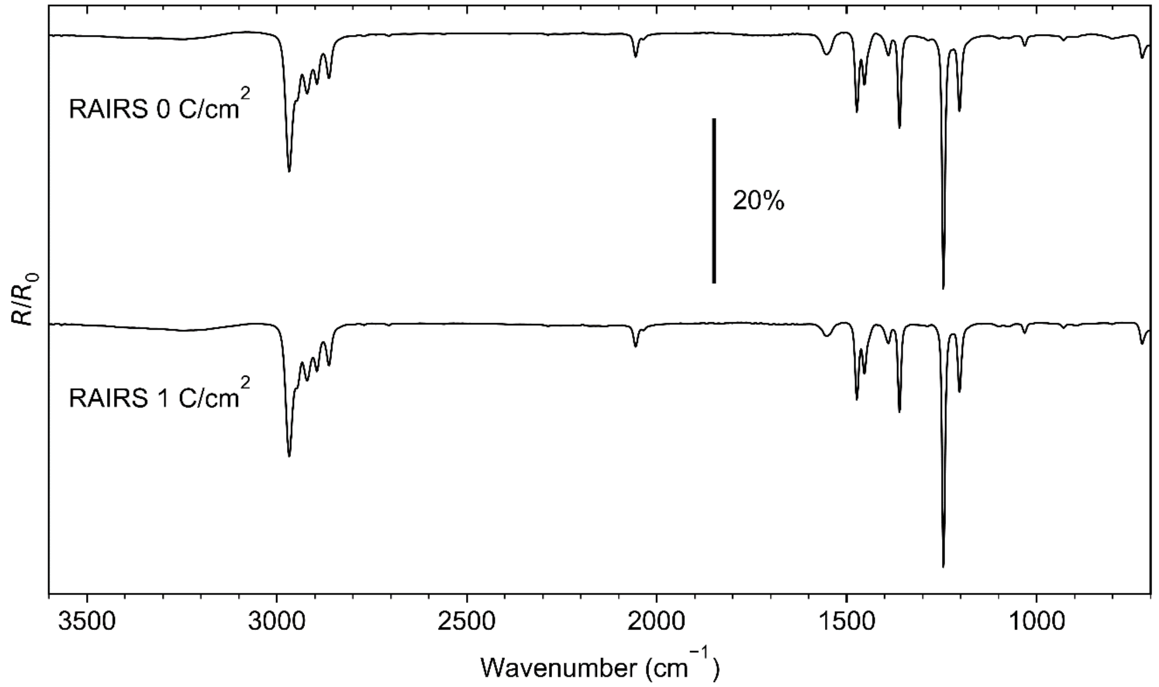

Figure S12. RAIR spectra of Ag(I) 3,3-dimethyl-1-butynyl before (0 C/cm^2) and after irradiation (1 C/cm^2) with E_0 = 100 eV.

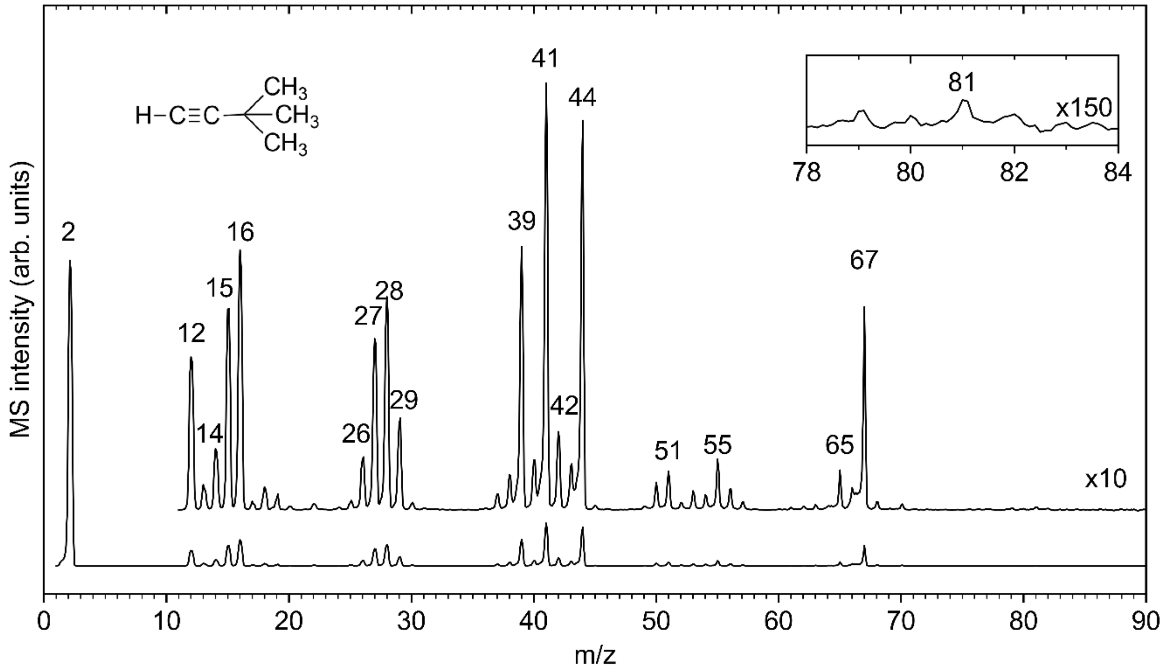

Figure S13. ESD mass spectra of the volatile species produced upon electron irradiation at 100 eV from a thick sublimate layer of Ag(I) 3,3-dimethyl-1-butynyl as also shown in Figure S12.

6. ESD kinetic experiments

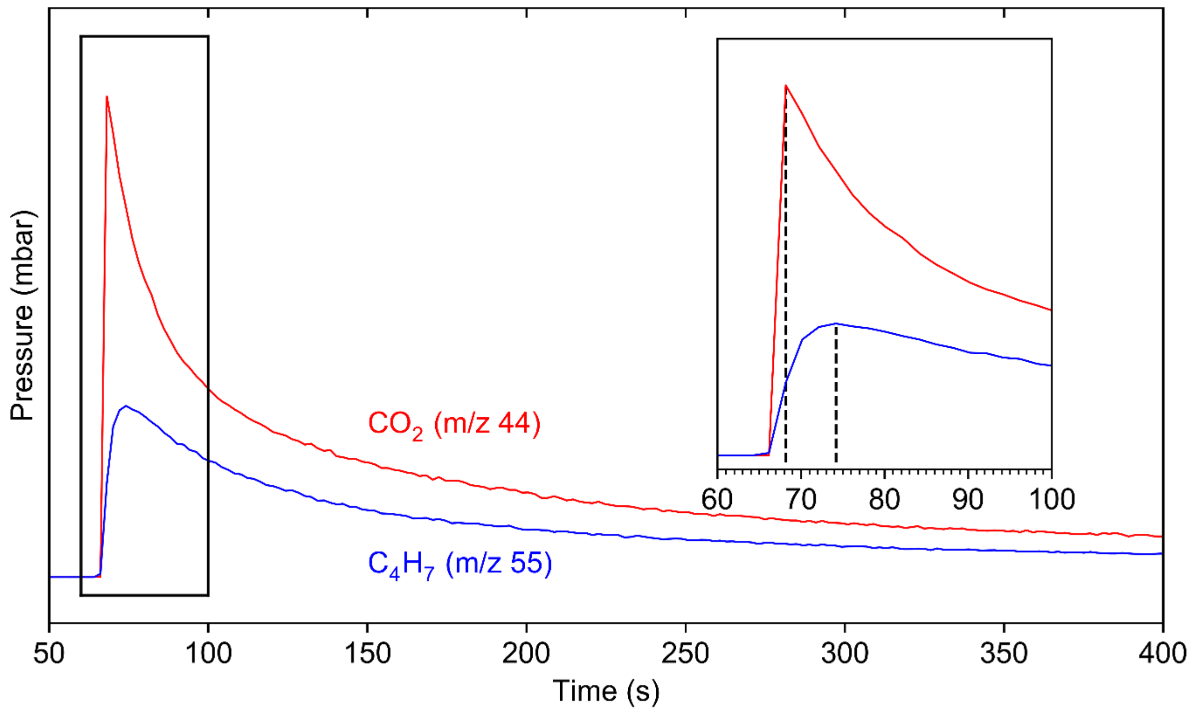

Figure S14. ESD as function of time during electron irradiation of Ag(I) 2,2-dimethylbutanoate at E_0 = 500 eV for m/z 44 (red, representative of CO_2) and m/z 55 (blue, representative of 2-methyl-2-butene). The sudden steep increase of the ESD signals marks the start of irradiation. During irradiation, the average current measured on the sample amounted to 1.4 µA/cm^2.

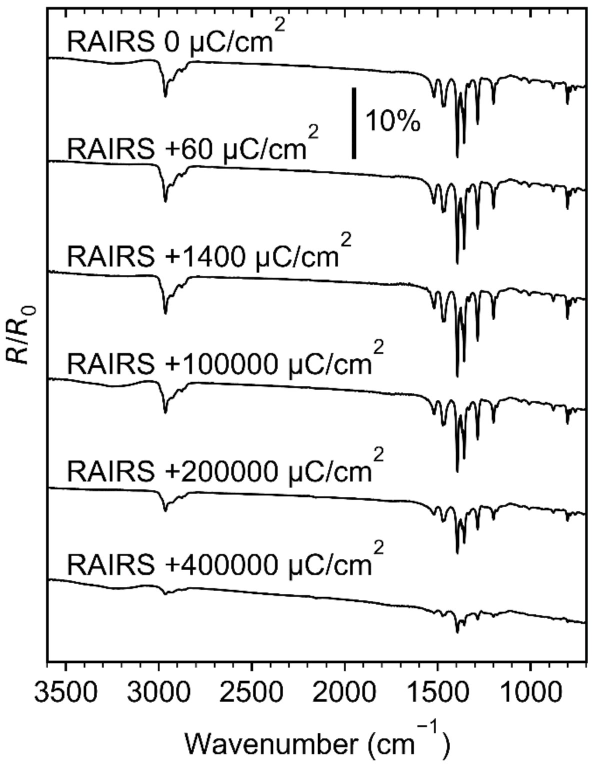

Figure S15. RAIRS of Ag(I) 2,2-dimethylbutanoate recorded before (0 C/cm^2) and after the electron exposures stated in the graph at an electron energy of 50 eV. The spectra were recorded during the experiment shown in Figure 9b of the main manuscript. Note that Figure 9b contains only the first three electron irradiation cycles after which ESD intensities were generally small.

References

1. Szymańska, I.; Piszczek, P.; Szczęsny, R.; Szłyk, E. Thermal and MS studies of silver(I) 2,2-dimethylbutyrate complexes with tertiary phosphines and their application for CVD of silver films. *Polyhedron* **2007**, *26*, 2440–2448.
2. Choi, H.J.; Han, S.W.; Lee, S.J.; Kim, K. Structure and thermal behavior of a layered silver hydroxyalkanecarboxylate. *J. Colloid Interface Sci.* **2003**, *264*, 458–466.
3. Lee, S.J.; Han, S.W.; Choi, H.J.; Kim, K. Structure and Thermal Behavior of a Layered Silver Carboxylate. *J. Phys. Chem. B* **2002**, *106*, 2892–2900.
4. Nelson, P.N. Chain Length and Thermal Sensitivity of the Infrared Spectra of a Homologous Series of Anhydrous Silver(I) n-Alkanoates. *Int. J. Spectrosc.* **2016**, *2016*, 1–9.
5. Ohe, C.; Ando, H.; Sato, N.; Urai, Y.; Yamamoto, M.; Itoh, K. Carboxylate−Counterion Interactions and Changes in These Interactions during Photopolymerization of a Long-Chain Diacetylene Monocarboxylic Acid at Air−Water Interfaces: External Infrared Reflection Absorption Spectroscopic Study. *J. Phys. Chem. B* **1999**, *103*, 435–444.
6. Lopez-Ramirez, M.R.; Ruano, C.; Castro, J.L.; Arenas, J.F.; Soto, J.; Otero, J.C. Surface-Enhanced Raman Scattering of Benzoate Anion Adsorbed on Silver Nanoclusters: Evidence of the Transient Formation of the Radical Dianion. *J. Phys. Chem. C* **2010**, *114*, 7666–7672.
7. Lewandowski, W.; Barańska, H. Comparison of the influence of silver, iron(III) and chromium(III) on the aromatic system of benzoic and salicylic acids in hydrated and anhydrous complexes. *Vib. Spectrosc.* **1991**, *2*, 211–220.
8. Green, J.; Kynaston, W.; Lindsey, A.S. The vibrational spectra of benzene derivatives—I. *Spectrochimica Acta* **1961**, *17*, 486–502.
9. Rintoul, L.; Shurvell, H.F. Raman and infrared spectra of solid 2,3-dimethylbutyne. *J. Raman Spectrosc.* **1998**, *29*, 791–798.
10. Jensen, J.O. Vibrational frequencies and structural determinations of tert-butylacetylene. *Vib. Spectrosc.* **2002**, *30*, 191–201.
11. Ramer, G.; Lendl, B. Attenuated Total Reflection Fourier Transform Infrared Spectroscopy. In *Encyclopedia of Analytical Chemistry*; Meyers, R.A., Ed.; John Wiley & Sons, Ltd: Chichester, UK, 2006, ISBN 9780470027318.
12. Ahlenhoff, K.; Koch, S.; Emmrich, D.; Dalpke, R.; Gölzhäuser, A.; Swiderek, P. Electron-induced chemistry of surface-grown coordination polymers with different linker anions. *Phys. Chem. Chem. Phys.* **2019**, *21*, 2351–2364.
13. Schrader, I.; Wittig, L.; Richter, K.; Vieker, H.; Beyer, A.; Gölzhäuser, A.; Hartwig, A.; Swiderek, P. Formation and structure of copper(II) oxalate layers on carboxy-terminated self-assembled monolayers. *Langmuir* **2014**, *30*, 11945–11954.
14. Danilov, V.; Wagner, H.-E.; Meichsner, J. Modification of Polydimethylsiloxane Thin Films in H_2 Radio-frequency Plasma Investigated by Infrared Reflection Absorption Spectroscopy. *Plasma Process. Polym.* **2011**, *8*, 1059–1067.
15. Streubel, P.; Hesse, R.; Makhova, L.; Schindelka, J.; Denecke, R. A Practicable Method for Thickness Estimation of Ultrathin Layers from XPS Data with UNIFIT 2011. Available online: http://www.unifit-software.de/PDF/Technical_Report_Thickness_Estimation.pdf (accessed on 10 March 2022).
16. Powell, C. *NIST Electron Effective Attenuation Length Database, NIST Standard Reference Database 82*, 2001.
